# Rutin Nanoparticles Alleviate Cadmium-Induced Oxidative and Immune Damage in Broilers’ Bursa of Fabricius via Modulating Hsp70/TLR4/NF-κB Signaling Pathway

**DOI:** 10.1007/s12011-024-04199-0

**Published:** 2024-05-04

**Authors:** Mohamed Abomosallam, Basma M. Hendam, Zeinab Shouman, Rasha Refaat, Nada M. A. Hashem, Shimaa A. Sakr, Noha M. Wahed

**Affiliations:** 1 Department of Forensic Medicine and Toxicology, Faculty of Veterinary Medicine, Mansoura University, Mansoura, 35516 Egypt; 2 Department of Animal Wealth Development, Faculty of Veterinary Medicine, Mansoura University, Mansoura, 35516 Egypt; 3 Department of Cytology and Histology, Faculty of Veterinary Medicine, Mansoura University, Mansoura, 35516 Egypt; 4 Phytochemistry and Plant Systematics Department, National Research Center, Dokki, Giza, 12622 Egypt; 5 Department of Physiology, Faculty of Veterinary Medicine, Mansoura University, Mansoura, 35516 Egypt

**Keywords:** Cd, Rutin, Nanoparticles, Immunotoxicity, TLR4, Hsp70, Broilers

## Abstract

Cadmium (Cd) is a serious environmental pollutant affecting various tissues/organs in broilers and compromising their immunological function and productivity. Therefore, the current study aimed to investigate Cd-induced immunotoxicity and potential immunoprotective effect of rutin nanoparticles (RNPs) in the bursal tissue of broilers. A total number of 150 chicks from the Hubbard breed were randomly divided into 5 groups. Group I was fed on standard basal diet (SD) with normal drinking water (DW), Group II received SD containing RNPs (50 mg/kg feed) with DW, Group III fed on SD and DW containing Cd (150 mg/L), Group IV co-treated with rutin-enforced SD (50 mg/kg diet) and DW containing Cd (150 mg/L), and finally, Group V co-supplemented with RNP-enhanced SD (50 mg/kg diet) DW containing Cd (150 mg/L). Productive performance, economic efficiency, oxidative biomarkers, histopathological changes, and the expression level of TLR-4, HSP-70, caspase 3, NF-κB, Bcl-2, and Bax were assessed in the BF tissue. Cd led to severe production and economic losses in exposed birds with a marked surge of oxidative biomarkers, pro-inflammatory cytokines, and histopathological changes in the bursal tissue which could be explained through upregulation of the Hsp70/TLR4/NF-κB molecular pathway in the BF tissue. Meanwhile, RNPs could alleviate most of these changes and prevail optimistic immunomodulatory properties which subsequently could enhance broilers’ productivity when incorporated in their diets.

**Graphical Abstract:**

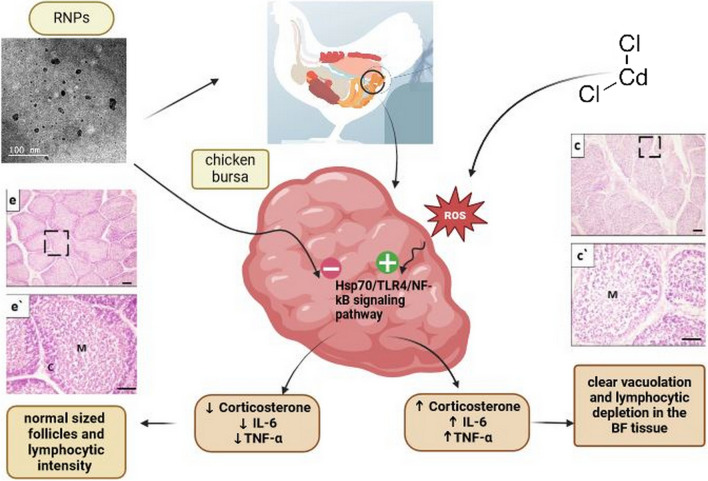

**Supplementary Information:**

The online version contains supplementary material available at 10.1007/s12011-024-04199-0.

## Introduction

Cadmium (Cd) is a poisonous heavy metal and released into the environment continuously from natural and industrial sources [1]. Exposure to small amounts of Cd over an extended period may produce serious effects in human and animal health [2]. The dietary intake of Cd in birds is implicated in various deleterious effects, including anemia [3], cardiotoxicity [4], hepatotoxicity [5], nephrotoxicity [6], and immunotoxicity [7]. Cd exerts its toxicity through excessive generation of free radicals with subsequent damage to various organs and tissue resulting in higher mortality rates and critical impacts upon animal productivity and economic efficiency [8, 9].

The immune system is a nexus between the organism and its environment, so environmental pollutants, including Cd, can cause serious damage to different components of immune system in broilers [10, 11]. Previous reports have indicated that Cd is an immunotoxic substance which not only affects innate immunity but also suppresses induced immunity leading to alteration of the overall immune function [12, 13]. Cd can also trigger apoptosis in the bursa of Fabricius (BF), which is the main immune organ in chickens, disrupt cytokine production, and provoke severe histopathological damage in the bursal tissue through excessive production of reactive oxygen species (ROS) leading to oxidative damage and several chronic illnesses [14–17]. Although Cd immunotoxicity in broilers has drawn more attention recently, the underlying molecular mechanism and interaction between Cd and chicken BF is still vague [13].

Various attempts have been made to combat the hazardous effects of Cd including dietary supplementation with various antioxidants, vitamins, and herbal substances [18]. Flavonoids are natural polyphenolic phytochemicals extracted from various vegetables, fruits, and grains and display a vast range of biological and pharmacological activities such as antioxidant, anti-inflammatory, antimutagenic, and antibacterial effect [19, 20]. As one of the best known dietary flavonoids, rutin has several pharmacological activities including high antioxidant capacity and radical scavenging activity [21, 22]. However, rutin’s poor bioavailability, easy degradation, and low water solubility could restrict its therapeutic effects, and numerous efforts have been made to improve the physicochemical properties and oral availability of rutin via various drug delivery strategies such as nanoparticles, solvates, and cyclodextrin complexes [23–25].

The promising features of nanoparticles can be employed to prepare sustained and targeted delivery system for various therapeutic applications [26]. Chitosan alginate is considered one of the best known natural and biocompatible polymeric nanoparticles which was extensively employed recently to enhance water solubility, bioavailability, absorption, and biological efficacy of hydrophobic drugs such as rutin [27, 28]. Chitosan is derived from deacetylation of natural chitin which is a component of crustacean exoskeleton, while alginate is a natural anionic polymer extracted from brown seaweed, and both have numerous biomedical and pharmaceutical applications [29–31].

Therefore, the current study aimed to synthesize RNPs in a chitosan-alginate core–shell structure to improve its antioxidant activity, broilers’ profitability, and productivity, in addition to evaluation of its immunoprotective effect against Cd-induced immunotoxicity in broiler bursal tissue, finally, addressing the gaps in our understanding of the molecular interaction between Cd and component of the immune system in birds.

## Materials and Methods

### Materials

Rutin trihydrate 95% (CAS: 250,249–75-3, MW: 664.57) was purchased from Alfa Aesar (Thermo Fisher Scientific, Haverhill, USA), and chitosan (low molecular weight and 90% DA) and sodium alginate were obtained from SRL (Mumbai, India). Anhydrous calcium chloride and acetic acid were provided by Piochem (Giza, Egypt). All the used chemicals were of laboratory grade.

### Synthesis of RNPs

Rutin (20 mg/mL) was dissolved in an ethanolic solution (50%, v/v), and this solution was added in a dropwise manner into 100 mL of sodium alginate solution (0.06% w/v, pH = 4.9), then stirred for 2 h, followed by a dropwise addition of 7 mL of calcium chloride solution (0.2%, w/v) into sodium alginate with continuous stirring (500 rpm for 30 min.), and then sonicated in ultrasonic bath (Crest Ultrasonics Corp., USA) at a frequency of 25 kHz for 10 min; 100 mL of chitosan solution (0.15% in 1% acetic acid, w/v) was then added into the calcium alginate pregel with mechanical stirring (1000 rpm for 1 h). The resulting suspension was equilibrated overnight then washed and ultra-centrifuged at 14,000 rpm for 30 min for further analysis [32].

### Characterization of RNPs

#### Transmission Electron Microscopy (TEM)

The morphology of RNPs was examined through TEM, whereas the drop of sample solution was dispersed on a carbon-coated copper grid and then dried before examination [33].

#### Fourier Transform Infrared (FT-IR) Spectroscopy

The FTIR of samples (chitosan alginate, rutin, and RNPs) were measured on a FTIR spectrophotometer (Perkin-Elmer, Norwalk, CT, USA). Samples were mixed with KBr, and then, the spectra were recorded in the range of 4000–400 cm^−1^ resolution [34].

### Experimental Design, Birds’ Diet, and Management

**Fig. 1:**
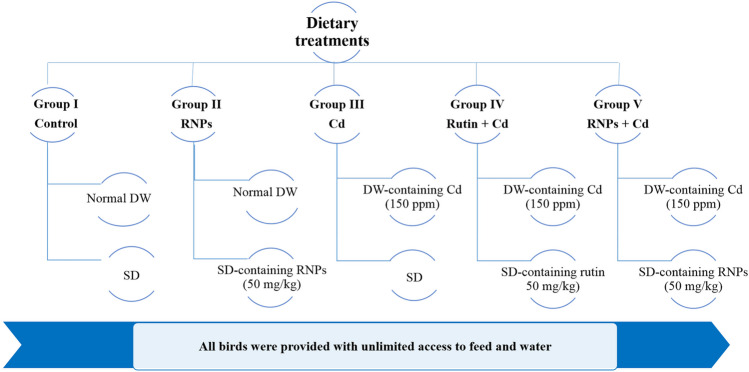
Schematic diagram for the dietary treatment

A total of 150 newly hatched Hubbard breed female chicks were obtained from a commercial hatchery (Alwatania Poultry Co., Mansoura, Egypt). On the 1st week, the initial temperature was maintained at around 32 °C and then reduced by 2 °C every 6–7 days until it reached 20 °C on day 35 till slaughtering. During the first few days, a lighting schedule of 23–24 h/day was applied to stimulate feeding and drinking, which later transitioned into a program of 12 h of light followed by 2 h of darkness. The basic diets as outlined in Table 1 (starter, grower, and finisher) were prepared based on the guidelines of the National Research Council [35]. After being adapted for a week, the birds were weighed, had their wings banded, and then, were distributed into five groups with three replicates each (30 birds/group, 10 birds/replicate) as illustrated in (Fig. 1). The five dietary treatments were as follows: Control group fed on SD (standard basal diet) and normal DW (drinking water), RNP-treated group fed on SD supplemented with 50 mg/kg RNPs based on previous studies [36, 37] and normal DW, Cd group fed on SD and 150 ppm cadmium chloride in DW according to previous reports [5, 9, 38, 39], rutin co-treated with Cd group fed on SD supplemented with rutin at 50 mg/kg and 150 ppm cadmium chloride in DW, and RNP co-treated with Cd group fed on SD enhanced with RNPs at 50 mg/kg and 150 ppm cadmium chloride in DW. All the birds were provided with unlimited access to feed and water, housed in well-ventilated rooms at a density of 10 birds/m^2^. Experimental protocol and methodology used in this study were approved in accordance with Mansoura University guidelines of the Animal Care and Use Committee (MU-ACUC), which strictly comply with the ARRIVE guidelines, under the approval No. VM.R.24.01.149. All efforts were made to reduce the number of animals and minimize their suffering. All experimental animals were euthanized by decapitation which was performed by a trained individual to ensure complete bleeding out of birds and minimize the birds’ suffering based on [40]. All chicks received vaccinations for Gumboro and Newcastle diseases in accordance with the guidelines provided by the manufacturer.

**Table 1:** Ingredients and chemical composition of basal diets

Ingredients	Starter (0–10 ds)	Grower (11–26 ds)	Finisher (27–40 ds)
Yellow corn	54.61	58.82	62
Soybean meal (46%)	36	33.9	29.93
Soybean oil	2.5	3.5	4
Corn gluten meal	2	0	0
Dicalcium phosphate	1.8	1.75	1.4
Limestone	1.46	1.36	1.2
L_Lysine	0.32	0.29	0.2
Sodium chloride	0.32	0.31	0.25
Vitamins and mineral premix	0.3	0.31	0.3
DL-methionine	0.25	0.26	0.27
Sodium bicarbonate	0.18	0.19	0.15
Anti-coccidian	0.05	0.05	0.05
Anti-mycotoxin	0.05	0.05	0.05
Anti-clostridia	0.03	0.03	0.03
L_Threonine	0.03	0.04	0
Energy enzyme	0.02	0.01	0.01
Lysomax	0.01	0.03	0.03
Choline chloride	0.05	0.05	0.05
Protease	0.01	0.04	0.02
Phytase enzyme	0.01	0.01	0.01
Chemical composition			
Dry matter (%)	89.64	89.59	89.51
Crude fiber (%)	2.28	2.23	2.17
Crude protein (%)	23.12	21.01	19.04
Crude fat (%)	4.94	5.99	7.01
ME (kcal/kg)	3050.95	3130.02	3210.87
Ash (%)	3.19	3.45	2.80
Lysine (%)	1.35	1.25	1.09
Sodium (%)	0.18	0.17	0.17
Methionine (%)	0.63	0.60	0.52
Calcium (%)	1.05	0.96	0.85
Available phosphorus (%)	0.50	0.46	0.42

Diet calculated according to National Research Council guidelines, supplied per kilogram of diet: vitamin D3, 2200 IU; vitamin E, 26 IU; vitamin A, 12,000 IU; vitamin K3, 6.25 mg; vitamin B2, 6.6 mg; vitamin B6, 1.5 g; vitamin B1, 3.75 mg; pantothenic acid, 18.8 mg; folic acid, 1.25 mg; biotin, 0.6 mg; vitamin B12, 0.31 mg; niacin, 30 mg; Se, 0.20 mg; Zn, 50 mg; Mn, 60 mg; Cu, 6 mg; Fe, 50 mg; I, 1 mg; and Co, 1 mg

*ME*, metabolic energy

### Productive and Economic Performance Evaluation

#### Productive Performance

The average body weight and feed intake (FI) of chicks were recorded throughout the study. The initial BW was measured after a week of adaptation, and the final weight was recorded at the end of the study. Furthermore, the feed conversion ratio (FCR) and mortality rates were also measured based on [41, 42].

#### Economic Evaluation Measures

The economic evaluation was conducted using the prevailed market price of chicks, diets, and live BW during the experiment in Egyptian pounds (LE). Total feed cost (FC), total cost of production (TC), total returns (TR), and net profit (NP) parameters were estimated based on previous studies [43, 44].

### Tissue Sample Collection

At the end of the experiment (40 D), 45 birds were randomly selected from five groups as nine birds per treatment (three birds per replicate), then weighed individually and slaughtered. BF tissue samples were washed and divided into three parts for evaluation of histopathological changes (fixed in 10% formaldehyde solution, pH 7.2), oxidative biomarkers (homogenized in 10% ice-cold phosphate buffer saline, pH 7.4), and molecular analysis (stored at − 80 °C). Furthermore, blood samples were collected from the jugular vein for hematological and biochemical studies, then centrifuged (3000 rpm for 10 min) and sera collected in sterile tubes to be processed for the respective biochemical investigations.

### Assessment of Oxidative Stress Biomarkers

BF tissue homogenate was centrifuged for 10 min at 3000 rpm, and then, the supernatant was collected and stored at − 80 °C for further biochemical analysis. The levels of MDA, GSH, CAT, SOD, and GPx were estimated via commercial diagnostic kits (Biodiagnostic, Egypt) following the manufacturer’s protocols with a UV-1240 Spectrophotometer (Shimadzu, Japan).

#### Return Function (Relation Between Total Return and Oxidative Stress Activity)

To evaluate the impact of oxidative stress system on broiler production TR, a return function was utilized. The logarithmic model of the function, as proposed by [45, 46], was employed for this purpose. Statistical tests, including the *t*-test, were used to determine the significance of relationships between TR and its influencing variables [47]. Additionally, the adjusted regression coefficient *R*^2^ was also calculated [48, 49].

### Biochemical and Hematological Analysis

The serum concentrations of corticosterone (CORT), interleukin-6 (IL-6), and tumor necrosis factor α (TNF-α) were quantified with commercial ELISA kits (CSB-EQ027342CH, CSB-E08549Ch, CSB-E11231Ch, respectively) (Cusabio Biotech Co., Wuhan, Hubei, China) based on the manufacturer’s protocol. Samples were measured in triplicates according to [50]; briefly, 100 μL of anti-chicken CORT, IL-6, and TNF-α monoclonal antibodies were added to microwell plate overnight at 4 °C. After incubation, standards and samples were added at room temperature and shaken for 2 h, followed by addition of biotinylated anti-chicken CORT, IL-6, and TNF-α antibodies for 1 h. Then, wells were washed, and avidin-HRP conjugate was incubated for 30 min before adding TMB substrate for 15 min. The optical density value was recorded at 450 nm on a microplate reader (Thermo Fisher, USA), then expressed as pg/mL. The detection range is between 15.6 and 1000 pg/mL for IL-6 and 0.27–200 pg/mL for TNF-α.

Concerning hematological parameters, blood samples were collected in EDTA tubes, and then, the total leukocytic count (TLC) was analyzed via a hematology analyzer (Bayer-Advia 120). For differential leukocytic count, a drop of blood was smeared on a glass slide for each bird and then stained through May-Grünwald and Giemsa stains. One hundred leukocytes including heterophils, lymphocytes, eosinophils, and monocytes were counted on the slide, and the heterophil-to-lymphocyte (H/L) ratio was also calculated based on [51, 52].

### Molecular Analysis

#### RNA Extraction and cDNA Synthesis

RNA was isolated from the BF tissue using the RNeasy Mini Kit (iNtRON Biotechnology, Seongnam, Korea) following the guidelines of the manufacturer’s procedures. The quality of the extracted RNA was qualified by 1.5% agarose gel electrophoresis. The extracted RNA was subsequently reverse transcribed using the Reverse Transcription kit (Qiagen, Heidelberg, Germany) from each sample based on the manufacturer’s guidelines. Then, cDNA samples were stored at − 20 °C until qRT-PCR.

#### qRT-PCR Analysis

Specific primer sequences of TLR4, HSP70, and caspase 3 genes beside their appropriate NCBI GenBank accession numbers are presented in Table 2. In brief, the qPCR investigation was performed in a Rotor-Gene Q apparatus with a QuantiTect® SYBR® Green PCR kit (SensiFast™ SYBR Lo-Rox kit, London, UK). The thermal cycling conditions of Rotor-Gene Q apparatus were 95 °C for 10 min, followed by 40 cycles of 95 °C for 15 s, 60 °C for 15 s, and 72 °C for 15 s. The melting-curve analysis was complete to confirm the specificity of the qPCR. The relative expression profile of the target genes was measured through the comparative 2 − ∆∆Ct method using GAPDH as a housekeeping gene for target gene standardization [53].

**Table 2:** Forward and reverse primer sequences used for RT-qPCR analysis

Gene	Primer sequence	Genbank accessation no	Product size (bp)	Reference
*TLR-4*	F:5′-GTTCCTGCTGAAATCCCAAA-3′ R:5′-TATGGATGTGGCACCTTGAA-3′	NM_001030693	133	Lu et al. (2014)
*HSP70*	F:5′-CCAAGAACCAAGTGGCAATGAA-3′ R:5′-CATACTTGCGGCCGATGAGA-3′	EU747335	72	Abdo et al. (2017)
Caspase 3	F:5′-AAAGATGGACCACGCTCAGG-3′ R:5′-TCCGGTATCTCGGTGGAAGT-3′	NM_204725.1	189	Reno et al. (2022)
*GAPDH*	F:5′- TCTTCACCACCGCTCAGTTC-3′ R:5′-TATCAGCCTCTCCCACCTCC-3′	NM_204305.1	114	Lu et al. (2014)

### Histopathological and Immunohistochemical Examination

BF tissues were fixed in neutral buffered formalin, then paraffin-embedded and sectioned (4 µm thickness). The sections were stained following deparaffinization with hematoxylin–eosin (H&E) method for microscopic examination [54]. Evaluation of immunohistochemical expression of nuclear factor-kappa B (NF-κB), Bax, and Bcl-2 in the bursal tissue was carried out following the indirect avidin–biotin-peroxidase staining technique based on [55]. Sections were incubated overnight at 4 ℃ with diluted primary antibodies against NF-κB (Cat# sc-8008), Bcl-2 (#sc-7382), and Bax (#sc-20067) (Santa Cruz Biotechnology, CA, USA). After the addition of the avidin–biotin complex, antibody binding was visualized with diaminobenzidine (DAB). All slides were observed and photographed through BX51 Olympus microscope with a built-in camera (Olympus optical LTD, Japan). Random five fields per slide were evaluated by mean of optical density using Image-Pro Plus 6.0 software (Media Cybernetics, MD, USA).

### Statistical Analysis

All data were displayed as mean ± standard error mean (SEM) and then analyzed using one-way ANOVA followed by Tukey’s post hoc test through SPSS statistical program software version 22 (SPSS Inc., Chicago, USA) in order to establish multiple comparisons between the control and treated groups at the same points of time [56]. Statistical significances between control, Cd, and RNP-treated groups were indicated at *p* ≤ 0.01.

## Results

### Characterization of RNPs

**Fig. 2:**
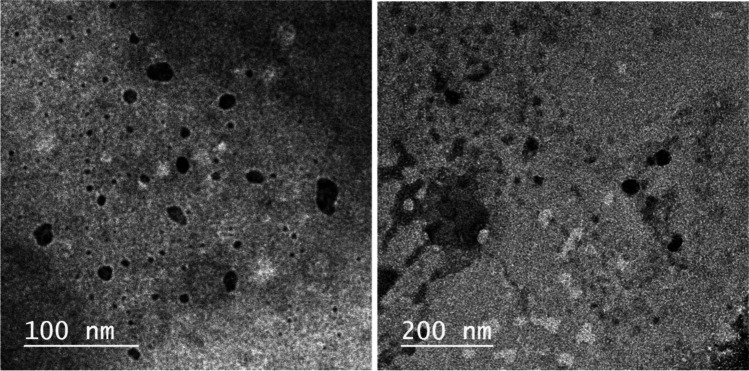
TEM images of RNPs (100 and 200 nm)

RNP morphology was investigated by TEM analysis which revealed nearly spherical particles with average particle size between 30 and 50 nm (Fig. 2).

**Fig. 3:**
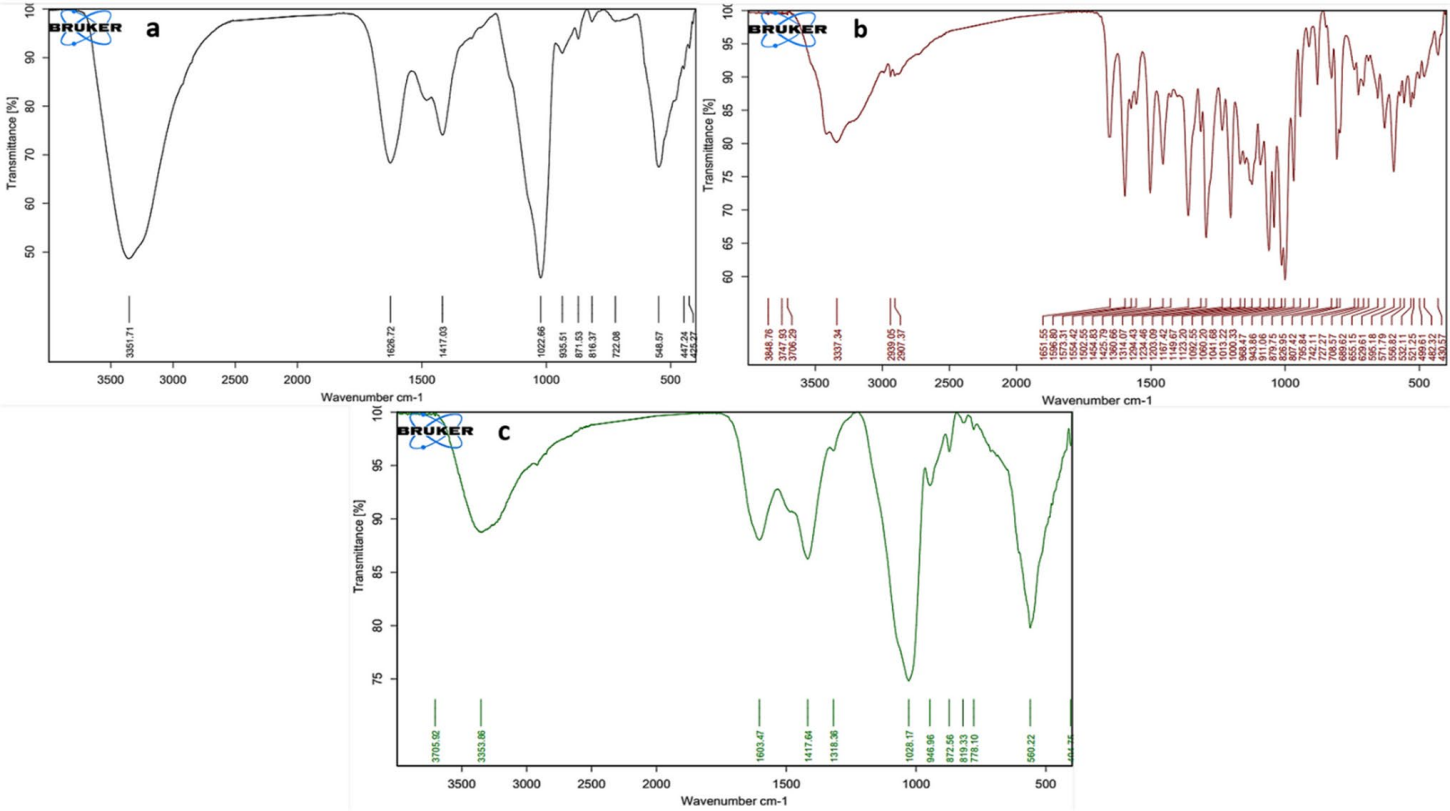
FTIR spectra of chitosan alginate biopolymer (**a**), rutin (**b**), and RNPs (**c**)

FTIR analysis of RNPs and its individual components, rutin and chitosan alginate polymer, could provide further information about RNP synthesis (Fig. 3). The FTIR spectra of chitosan alginate polymer revealed its characteristic peaks through an interaction between amino groups of chitosan (positively charged) and carboxyl groups of alginate (negatively charged) at 1626 (N–H bending of primary amine and symmetric COO − stretching), 1417 (C-N stretching of amide group and asymmetric COO − stretching), 1022 (C-O stretching), and 3351 (O–H stretching vibration). Pure rutin, a polyphenolic compound, in the FTIR spectrum exhibited spectral peaks at 3337 and 2907 cm^−1^ (O–H and C-H stretching of phenols, respectively), 1651 and 1502 cm^−1^ (C = O and C = C stretching of carboxyl groups), and 1360 and 1294 cm^−1^ (C-O stretching of phenolic groups). Other characteristic peaks were displayed between 1203 and 1000 cm^–1^ (C–O–C and C–OH stretching) which supported the presence of phenolic and carboxylic groups of rutin. In the case of RNPs, the IR spectrum revealed certain changes in intensity and position of peaks compared with other IR spectra. For example, the characteristic peak of chitosan alginate polymer at 1626 broadened and shifted from 1626 to 1603, besides the appearance of a new peak at 1318 which confirms the successful loading of rutin into chitosan alginate nanocomposite.

### Productive Performance

Table 3 illustrates the effect of rutin and RNPs on growth performance against Cd-induced immunological and oxidative stress in broiler chickens. Broilers treated with RNPs showed significantly better growth performance compared to the other groups since the final BW and BWG increased by 7.17% and 9.63%, respectively, in comparison to the control group. In addition, those broilers showed the best FCR, which decreased by 9.32% compared to the control group. On the contrary, the Cd-intoxicated group exhibited the poorest productive performance based on the final BW and BWG, which decreased by 39.17% and 53.49%, respectively, compared to the control group. Additionally, FCR of this group was increased by 112.05% compared to the control group.

**Table 3:** Effect of rutin and RNPs on growth performance and economic parameters against Cd-induced immunological stress in broiler chickens

Item/bird	Control	RNPs	Cd	Cd + rutin	Cd + RNPs
Initial BW (g)	794.17 ± 4.74	797.50 ± 3.36	795.50 ± 4.21	798.00 ± 2.79	795.50 ± 1.73
Final BW (g)	2975.0^b^ ± 10.17	3188.3^a^ ± 3.59	1809.7^e^ ± 5.23	2883.0^d^ ± 7.02	2925.0^c^ ± 13.47
BWG (g)	2180.8^b^ ± 12.26	2390.8^a^ ± 4.37	1014.2^e^ ± 5.47	2085.0^d^ ± 9.39	2129.5^c^ ± 13.84
Total FI (g)	4870.0 ± 17.15	4836.7 ± 33.64	4803.3 ± 31.6	4855.0 ± 27.68	4825.0 ± 36.05
FCR	2.24^ cd^ ± 0.06	2.02^c^ ± 0.05	4.75^a^ ± 0.22	2.33^b^ ± 0.07	2.27^bc^ ± 0.06
Mortality (%)	13.33 ± 3.33	6.67 ± 3.33	16.67 ± 3.33	10.00 ± 5.77	6.67 ± 3.33
FC (LE)	102.27^b^ ± 0.36	108.1^a^ ± 0.71	100.87^b^ ± 0.67	107.96^a^ ± 0.58	107.83^a^ ± 0.76
TC (LE)	131.27^b^ ± 0.36	137.1^a^ ± 0.71	129.87^b^ ± 0.67	136.96^a^ ± 0.58	136.83^a^ ± 0.76
TR (LE)	201.33^a^ ± 0.51	209.79^a^ ± 0.18	121.97^b^ ± 0.26	195.16^a^ ± 0.35	197.98^a^ ± 0.67
NP (LE)	70.1^a^ ± 0.53	72.69^a^ ± 0.56	− 7.9^b^ ± 0.8	58.21^a^ ± 0.34	61.15^a^ ± 0.99

Values are displayed as mean ± SEM. The mean values with different small superscript letters within the same row differ significantly at *P* < 0.01

*BW*, body weight; *BWG*, body weight gain; *FI*, feed intake; *FCR*, feed conversion ratio; *FC*, feed cost; *TC*, total cost; *TR*, total return; *NP*, net profit; *LE*, Egyptian pound

Regarding co-treatment with rutin and RNPs after Cd intoxication, the rutin-co-treated group showed an increase in the final BW and BWG by 59.31% and 105.58%, respectively, when compared to the Cd-intoxicated group. Similarly, the RNP-co-treated group exhibited a significant rise in the final BW and BWG values by 61.63% and 109.97%, respectively, when compared to the Cd-intoxicated group. Furthermore, the FCR was markedly declined in rutin and RNP-co-treated groups by 50.95% and 52.21%, respectively, in comparison to the Cd-intoxicated group, while there was no significant change in the mortality rates between the control and different treated groups.

### Economic Evaluation

Table 3 demonstrates the economic evaluation of rutin and RNP treatment against Cd-induced immunological and oxidative stress in broiler chickens. Concerning feed cost, a significant difference (*P* < 0.01) between groups was recorded since the feed cost declined by 1.37% in the Cd-intoxicated group but increased by 5.7% in the group treated with RNPs only when compared to the control group. However, the feed costs markedly were elevated by 6.99% and 7.03% in rutin and RNP-co-treated groups, respectively, in comparison to the Cd-intoxicated group.

In terms of TC, the RNP-supplemented group revealed a notable rise in TC by 4.44% while the Cd-intoxicated group showed a relative decline in TC by 1.1% when compared to the control group. However, rutin and RNP-co-treated groups following Cd exposure showed a marked rise in TC by 5.46% and 5.36%, respectively, when compared to the Cd-intoxicated group.

Based on TR data, the group that received RNPs alone had the highest TR (209.79 LE/bird) with an increase in rate of 4.2% compared to the control group. In contrast, the Cd-intoxicated group had the lowest reported TR (126.17 LE/bird) with a lower proportion of 39.42% compared to the control group. Additionally, the groups co-treated with rutin and RNPs displayed enhanced TR by 60.01% and 62.32%, respectively compared to the Cd-intoxicated group.

Regarding NP, our data showed a significant difference (*P* < 0.01) between groups since the RNP-treated group experienced the highest NP value (72.69 LE/bird) which increased by 3.69% compared to the control group. In contrast, the Cd-intoxicated group showed the lowest NP value (7.9 LE/bird) that declined by 111.27% when compared to the control group. In contrast, the NP was significantly improved by about 836.84% and 874.05% in groups co-treated with rutin and RNPs, respectively, in contrary to the Cd-intoxicated group.

### Evaluation of Oxidative Stress Biomarkers

Cd-treated birds, as illustrated in Table 4, exhibited a significant rise in MDA level by 158% with a marked decline in non-enzymatic antioxidant GSH by 39.27% and enzymatic antioxidants including SOD, CAT, and GPx by 52.1%, 59.75%, and 48.25%, respectively, in BF tissue samples compared with the control group (*P* < 0.01). However, in contrary to the Cd-intoxicated group, rutin and RNP-co-treated groups displayed a notable decline in MDA level by 38.57% and 56.2%, respectively (*P* < 0.01). Moreover, there is an outstanding elevation of GSH, SOD, CAT, and GPx levels by 16.18%, 54.04%, 37.95%, and 27.96% in rutin-co-treated broilers and by 51.52%, 96.48%, 129.09%, and 75.88% in RNP-co-supplemented birds in comparison to the Cd-treated group (*P* < 0.01).

**Table 4:** Effect of rutin and RNPs on oxidative stress biomarkers, antioxidant enzymes, and return function against Cd-induced immunological stress in broiler chickens

Group	Control	RNPs	Cd	Cd + rutin	Cd + RNPs
MDA	33.33^d^ ± 0.88	30.17^d^ ± 0.48	86^a^ ± 0.52	52.83^b^ ± 0.7	37.67^c^ ± 0.61
GSH	27.17^ab^ ± 0.6	28.17^a^ ± 0.48	16.5^d^ ± 0.43	19.17^c^ ± 0.48	25^b^ ± 0.58
SOD	99.17^b^ ± 0.6	104.33^a^ ± 1.02	47.5^e^ ± 0.76	73.17^d^ ± 0.79	93.33^c^ ± 0.67
CAT	83.67^a^ ± 0.84	85.17^a^ ± 0.7	33.83^d^ ± 0.87	46.67^c^ ± 0.67	77.5^b^ ± 0.76
GPx	122.33^b^ ± 0.84	128.83^a^ ± 0.79	63.3^e^ ± 0.88	81^d^ ± 0.97	111.33^c^ ± 0.8
Return function (relation between TR and antioxidant enzymes as well as oxidative stress biomarkers)					
Log TR =	0.75 + 0.59 Log GPX + 0.61 log SOD + 0.43 log CAT + 0.67log GSH-0.44log MDA				
*t*-value	0.78^Ns^, 7.67**, 11.8**, 7.59**, 5.91**, − 0.87**				
*R*^2^	0.92				
*F*	66.33**				

Values are displayed as mean ± SEM; the mean values with different small superscript letters within the same row differ significantly at *P* < 0.01

*Ns*, non-significant; *TR*, total return

**Significant at *P* < 0.01

The return function which emphasized the relationship between TR and oxidative stress system, as displayed in Table 4, revealed that a 1% increase in GSH led to an approximate 0.67% increase in TR. Similarly, a 1% increase in SOD, CAT, and GPx enzymes corresponded to about 0.61%, 0.43%, and 0.59% increase in TR, respectively. Conversely, a 1% rise in MDA level caused a decline of approximately 0.44% in TR. Furthermore, the analysis of the return function revealed that approximately 92% of variations in the broiler farm’s TR can be attributed to alterations in oxidative stress system in broilers.

### Biochemical and Hematological Analysis

Cd-intoxicated birds revealed a substantial rise of serum corticosterone, TNF-α and IL-6 levels (*P* < 0.01) by 140.14%, 158.87%, and 50.72%, respectively, when compared to the control birds as summarized in Table 5. Meanwhile, co-treatment with rutin and RNPs significantly reduced CORT and TNF-α and IL-6 levels (*P* < 0.01) by 21.53%, 20.01%, and 16.4%, respectively, for rutin and by 44.19%, 49.25% and 29.64% correspondingly for RNPs when compared to the Cd-intoxicated group.

**Table 5:** Effect of rutin and RNPs on biochemical and hematological biomarkers against Cd-induced immunological stress in broiler chickens

Group	Control	RNPs	Cd	Cd + rutin	Cd + RNPs
CORT (ng/mL)	1.47^ cd^ ± 0.15	1.4^d^ ± 0.12	3.53^a^ ± 0.0.15	2.77^b^ ± 0.09	1.97^c^ ± 0.09
TNF-α (pg/mL)	41.23^d^ ± 2.19	39.37^d^ ± 1.16	106.73^a^ ± 1.9	85.37^b^ ± 1.27	54.17^c^ ± 1.55
IL-6 (pg/mL)	181.6^d^ ± 1.74	180.1^d^ ± 1.74	273.7^a^ ± 2.03	228.8^b^ ± 1.14	192.57^c^ ± 1.72
TLC (10^3^/μL)	20.83^ab^ ± 0.77	21.37^a^ ± 0.52	17.93^b^ ± 0.96	19.80^ab^ ± 0.35	20.60^ab^ ± 0.47
Heterophils (%)	23.1^c^ ± 1.28	22.1^c^ ± 1.23	37.23^a^ ± 0.78	31.83^b^ ± 0.83	25^c^ ± 1.44
Lymphocytes (%)	75.27^a^ ± 0.55	76.13^a^ ± 0.18	61.33^c^ ± 0.64	67.7^b^ ± 0.35	73.53^a^ ± 1.01
Monocytes (%)	1.83^a^ ± 0.09	1.9^a^ ± 0.06	2.1^a^ ± 0.12	1.87^a^ ± 0.15	1.9^a^ ± 0.12
H/L ratio	0.31^c^ ± 0.02	0.29^c^ ± 0.02	0.61^a^ ± 0.01	0.47^b^ ± 0.01	0.34^a^ ± 0.02

Values are displayed as mean ± SEM. The mean values with different small superscript letters within the same row differ significantly at *P* < 0.01

*CORT*: corticosterone, *TNF-α*: tumor necrosis factor α, *IL-6*: interleukin-6, *TLC*: total leukocytic count, *H/L ratio*: heterophil-to-lymphocyte ratio

For hematological biomarkers, Cd-intoxicated broilers also showed a significant decline in the TLC by 13.92% with a notable reduction in the lymphocyte percentage while the heterophile percentage and the H/L ratio were significantly elevated in comparison to the control group (*P* < 0.01) (Table 5). On the other side, co-treatment with rutin and RNPs enhanced the TLC by 10.43% and 14.89%, respectively, when compared to the Cd-intoxicated group (*P* < 0.01). The lymphocyte percentage was relatively increased, particularly in the RNP-co-treated group, beside a marked decline in the heterophile percentage and the H/L ratio in contrast to the Cd-intoxicated group.

### Quantitative RT-PCR Analysis

**Fig. 4:**
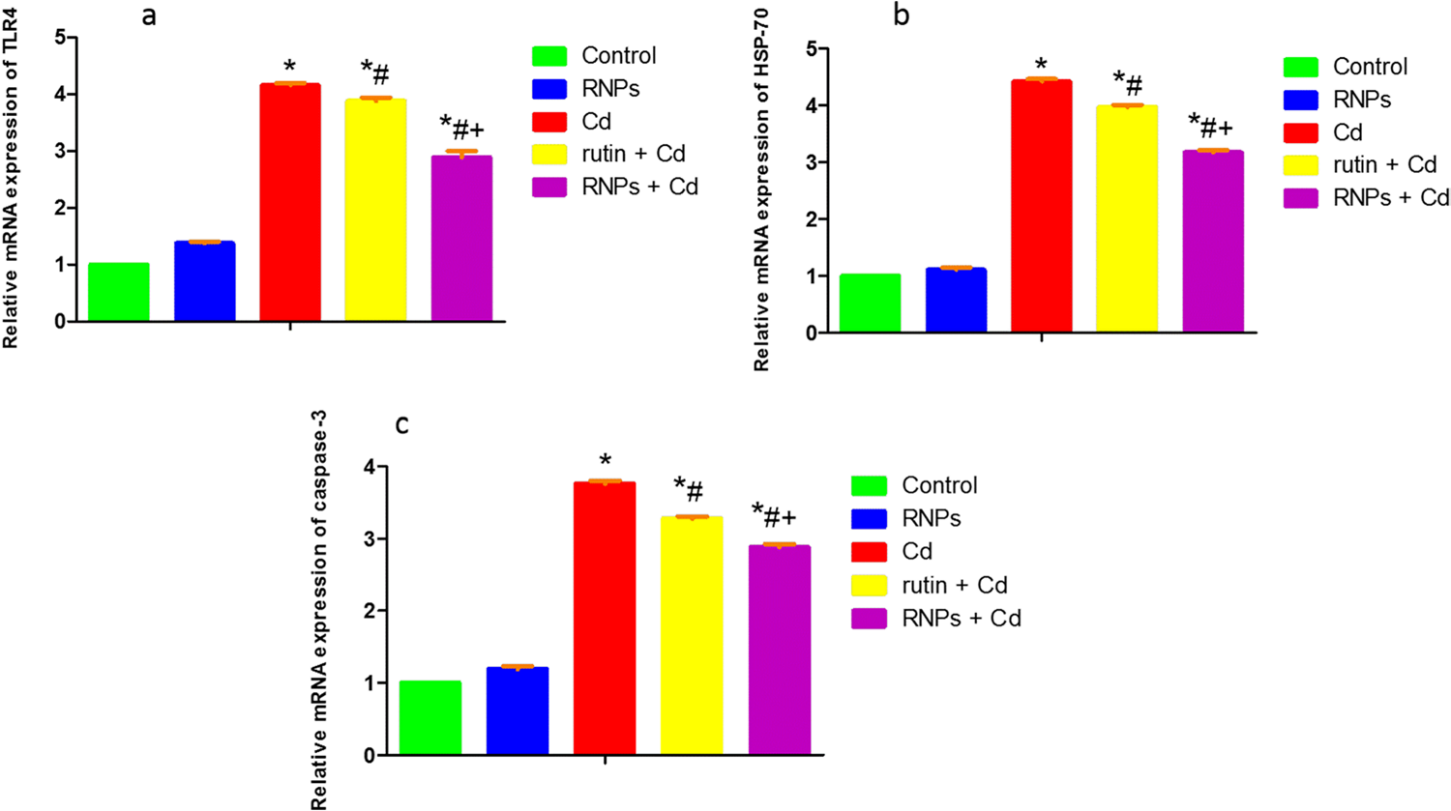
Relative mRNA expression of **a** TLR4, **b** HSP70, and **c** caspase 3 genes in the BF tissue of broilers treated with rutin and RNPs against Cd-induced immunological stress. Data expressed as mean ± SEM (*n* = 45) were analyzed via one-way ANOVA followed by Tukey’s test, and different letters indicate statistical significance at *P* < 0.01

The relative gene expression levels of TLR4, HSP70, and caspase 3 genes in BF tissue were illustrated in Fig. 4. Our data displayed that Cd treatment dramatically upregulated expression levels of TLR4, HSP70, and caspase 3 gene in the BF tissue by 316%, 342%, and 276%, respectively, when compared to the control group (*P* < 0.01). On the contrary, rutin and RNP co-supplementation significantly downregulated mRNA expression levels of TLR4, HSP70, and caspase 3 gene in the BF tissue by 6.73%, 10.18%, and 12.77%, respectively, for rutin and by 30.53%, 28.28%, and 23.4%, respectively, for RNPs in opposition to Cd-treated birds (*P* < 0.01).

### Histopathological and Immunohistochemical Evaluation

**Fig. 5:**
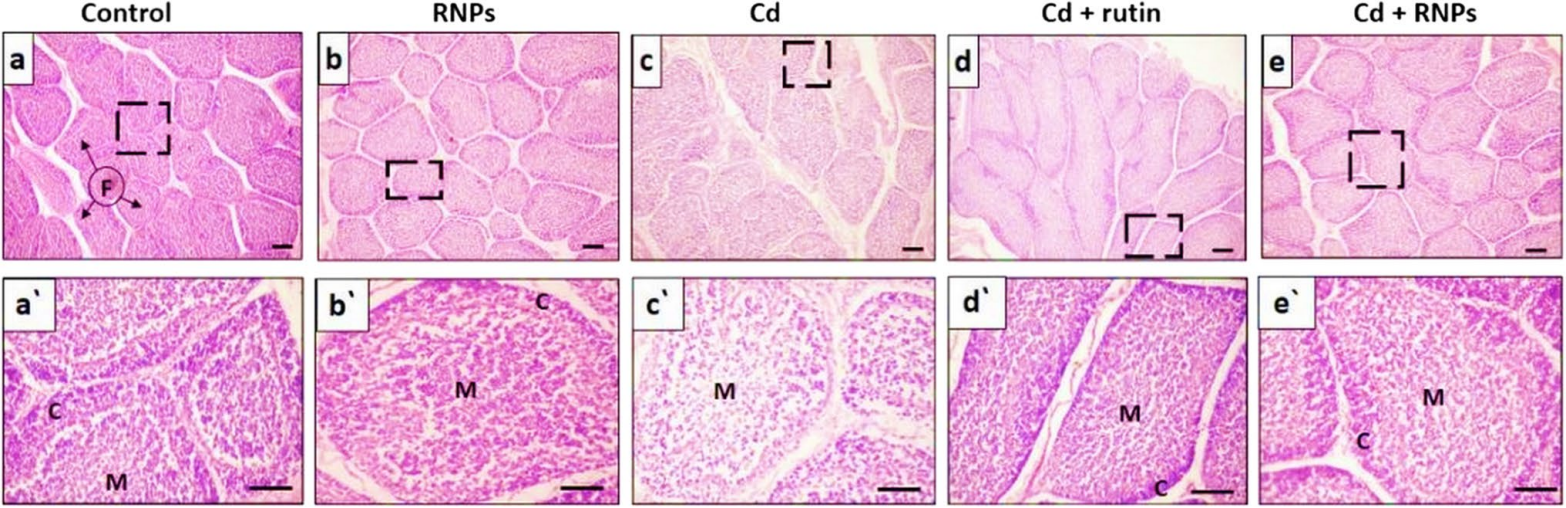
Photomicrograph of H&E-stained sections of BF tissue showing the control group with normal medullar and cortical structure (**a**, a’), RNPs group showed normal-sized follicles (**b**, b’), Cd group showed follicular atrophy with vacuolation, lymphocytic depletion, and interstitial connective tissue hyperplasia (**c**, c’), Cd + rutin group (**d**, d’) with relative lymphocytic depletion with reduction of the lymphocytic populations, and Cd + RNP group (**e**, e’) revealed a normal histological structure of the lymphoid follicles and nearly similar histological architecture to that of the control group. F, follicles; C, cortex; M, medulla. Scale bars = 100 µm and 50 µm for the magnified insets

The control and RNP groups showed a normal histological structure of the BF with normal-sized follicles. Each follicle exhibited two distinct regions (cortex and medulla) separated by a clear corticomedullary junction and had a normal lymphocytic intensity (Fig. 5a, a’, b, b’). In contrast, the Cd-treated group revealed a marked reduction in the number and size of follicles in the BF tissue with clear vacuolation and lymphocytic depletion (Fig. 5c, c’). The group co-treated with rutin showed a relative reduction of the lymphocytic populations (Fig. 5d, d’). Meanwhile, the birds co-supplemented with RNPs revealed nearly similar histological architecture to that of the control group (Fig. 5e, e’).

Additionally, the follicle number and size of the BF tissue were counted in five random fields. Data emphasized that the Cd-treated group displayed a significant decrease in the number and size of follicles compared to the control group (Fig. 7a, b). On the other hand, the group co-treated with RNPs exhibited a significant increase in follicular number and size compared to the Cd-treated group.

### Immunohistochemical Expression of NF-κB, Bax, and Bcl-2 in the BF Tissue

**Fig. 6:**
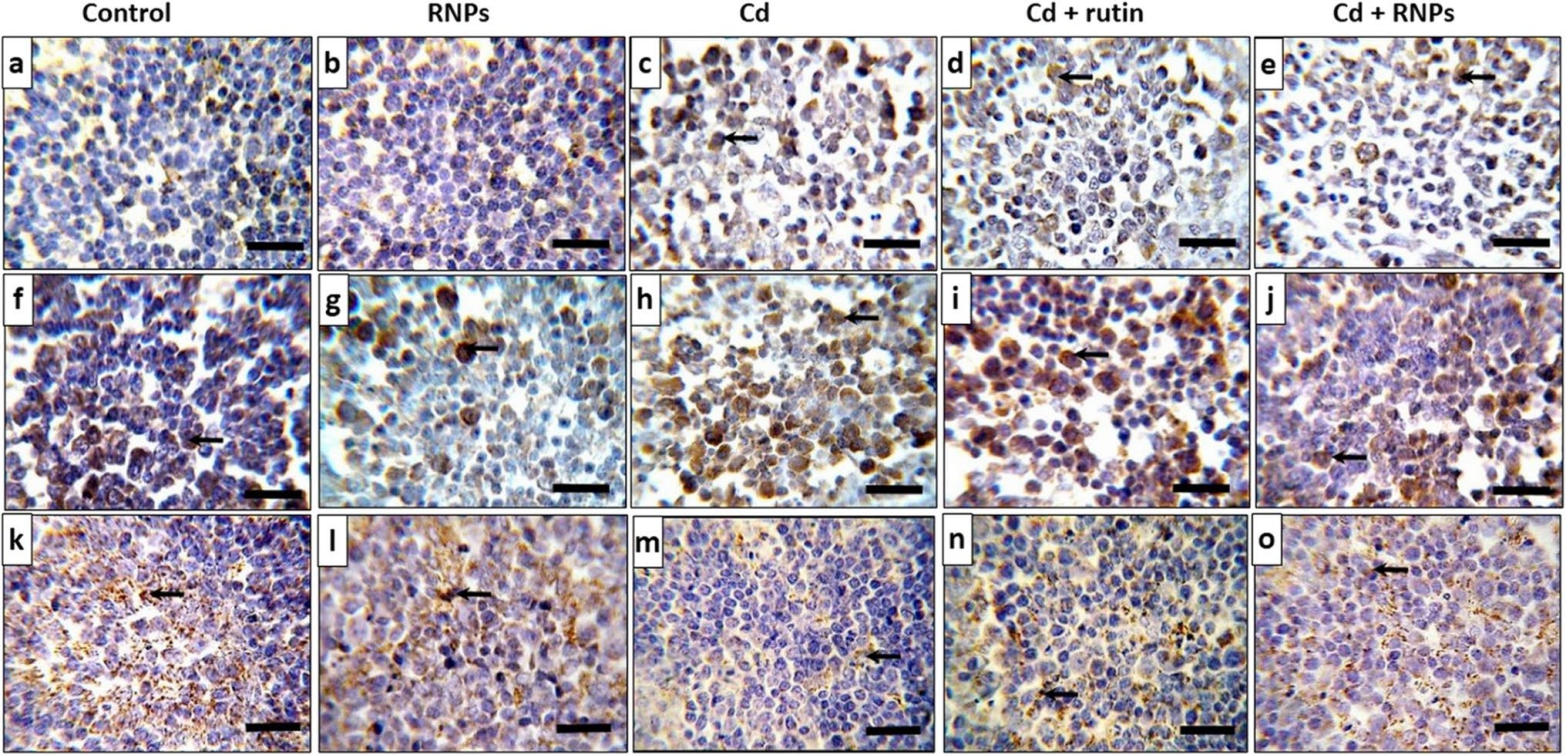
Photomicrograph of immunohistochemical staining of the BF tissue with anti-NF-κB (**a**–**e**) and anti-Bax (**f**–**j**) that revealed the minimal density of immunohistochemical staining in the control- and RNP-treated groups, while there was a marked increase in immunostaining in the BF tissue of Cd-treated birds. However, anti-Bcl-2 (**k**–**o**) showed a significant rise in the immunohistochemical staining in the control- and RNP-treated groups, and the density of immunohistochemical staining was minimal in Cd-treated groups. Positive cells show a brown color (arrows). Scale bars = 30 µm

**Fig. 7:**
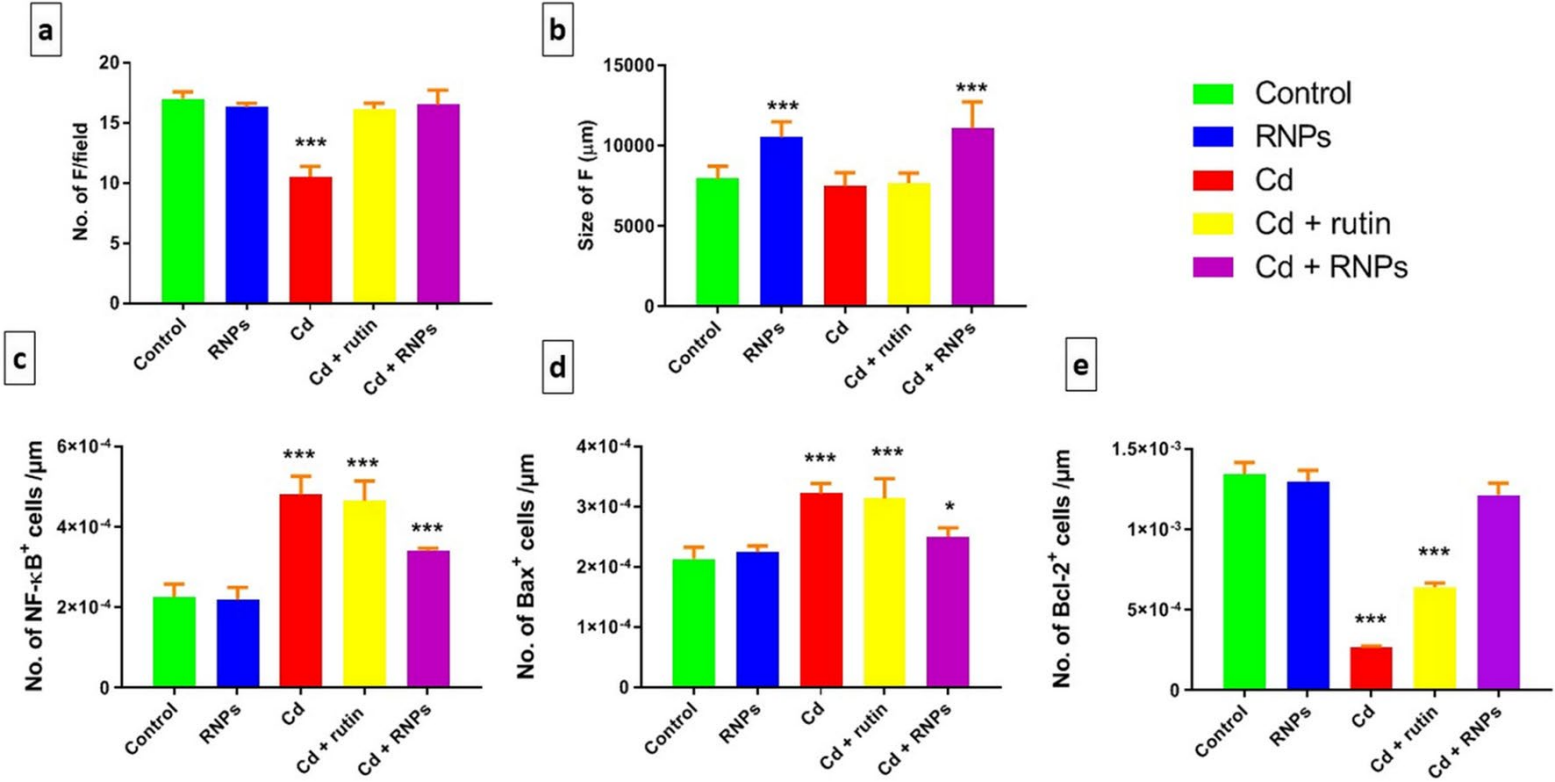
Graphs showing the estimated results of the number of the bursal follicles of all groups (**a**), size of follicles (**b**), number of NF-κB + cells (**c**) and number of Bax + cells (**d**), and number of Bcl-2 + cells (**e**) in the BF tissue following treatment with rutin and RNPs against Cd-induced immunotoxicity. Significant results are represented by asterisks (*P ≤ 0.05, ***P ≤ 0.01)

The immunohistochemical staining was performed to evaluate the expression of NF-κB, Bax, and Bcl-2 in bursal cells. Examination of the BF sections of the control and RNPs groups showed a marked decline of NF-κB and Bax immunoreaction in the bursal cells (Fig. 6a, b, f, g), respectively. Meanwhile, the BF sections of the Cd-treated group showed a notable rise of NF-κB and Bax immunoreaction in contrary to the control group (Fig. 6c, h), respectively. Rutin co-treated with Cd showed less prominent NF-κB and Bax immunohistochemical staining in comparison to the Cd-exposed group (Fig. 6d, i), respectively, while RNPs co-supplemented with the Cd group showed relatively similar immunoreaction to that of the control group (Figs. 6e, j and 7c, d). On the other hand, BF sections of the control and RNP groups revealed a notable increase in the Bcl-2 immunoreactivity when compared with the other treated groups (Fig. 6k, l), while the Cd-exposed group showed a substantial decrease of Bcl-2 immunoreaction in BF cells in opposition to the control group (Fig. 6m). The BF cells of the group co-supplemented with rutin and Cd displayed an increase in positive immunoreaction of Bcl-2 when compared with the Cd-treated group (Fig. 6n), while the group co-treated with RNPs showed a significant increase in Bcl-2 immunoreaction which resemble that of the control group (Figs. 6o and 7e).

## Discussion

The current study evaluates the antioxidant effect of RNPs against Cd-induced oxidative damage and immunosuppression in broiler chickens. Experimentally, our findings revealed that Cd could have a critical immunotoxic effect in broilers through substantial elevation of oxidative biomarkers and pro-inflammatory cytokines which may provoke degenerative and pathological changes in the BF tissue with marked reduction in the lymphocytic population. However, rutin mitigated some of those changes, and RNP displayed promising results with complete restoration of the normal function and antioxidant activity in the BF tissue.

Cd is a nonessential and toxic element that is released into the environment through extensive usage of industrial fertilizers, pigments, and plastics [57]. Cd could bioaccumulate among various food chain levels, including poultry, resulting in severe malfunction of multiple organs such as the liver, kidneys, and nervous and immune systems [58, 59]. Concerning the catastrophic drawbacks of Cd in the poultry industry, it leads to significant productive and economic losses, and therefore, a growing concern should be paid to such pollutants emerging in the production systems [60]. The main exposure source of Cd in broilers is through ingestion of contaminated drinking water or food which subsequently upsurges the free radical production causing oxidative lesions in various tissues [61].

Various therapeutic interventions against heavy metals are gaining great attention recently [62]. Rutin, as a phytochemical, has proven to have a powerful antioxidant activity to alleviate oxidative stress and could strengthen the birds’ immunity [63]. Rutin also has mucosal protection and antiulcer functions which in turn improve the broilers’ growth performance and intestinal function; however, the poor aqueous solubility of rutin could limit their efficacy [64–66]. Therefore, rutin was efficiently loaded on chitosan alginate polymeric nanoparticles which are natural, biocompatible, biodegradable, and safe drug delivery vehicle to improve its bioavailability, solubility, stability. and efficacy [32, 67].

TEM displayed that RNPs had spherical shapes, smooth surfaces, and small particle sizes which could enhance its permeability across the GIT, and these findings come in agreement with previous studies [68]. Furthermore, FTIR data confirmed the efficient loading of rutin upon the polymeric nanoparticles since the characteristic peak of chitosan alginate polymer at 1626 cm^−1^ was broadened and shifted to 1603 cm^−1^ beside the appearance of a new peak at 1318 cm.^−1^, and these findings are consistent with previous reports [26, 69].

Concerning productive and economic performance, our findings exhibited that Cd significantly reduced the BW of exposed chickens, possibly due to a decrease in the FI and appetite beside an increase in the breakdown of proteins and fats associated with Cd toxicity [70]. Additionally, the negative impact of Cd on performance may be linked to its toxic effects on various systems within the animal body [71]. On the contrary, a notable rise in the FCR was observed in Cd-intoxicated chickens which may be linked to the reduction in BWG. These findings were consistent with [72] who reported that Cd-exposed broilers at 100 ppm in drinking water for 28 days showed a marked decline in the BW, BWG, and FI. Similarly [73], concluded that Cobb chickens treated with Cd chloride at a dosage of 50 mg/kg displayed a substantial elevation in the FCR beside a noticeable decline in the BWG and FI.

On the flip side, dietary addition of rutin and RNPs could potentially enhance the productive performance in broilers which comes in agreement with [74] who indicated that flavonoids such as rutin could protect the intestinal mucus membrane and prevent ulcers via blocking the gastric proton pump which subsequently stimulate the birds’ appetite beside promoting a healthy balance of intestinal microenvironment [75]. Furthermore, our findings matched with previous studies as [76, 77] which suggested that rutin supplementation in broiler diets could significantly improve the BWG and FCR.

Concurrently, some varieties of flavonoids are degraded in the intestinal fluid and have limited absorption capability through the intestinal membranes, so encapsulation of these types of flavonoids in nanoparticulate form could enhance their absorption capacity and availability [78]. Chitosan is considered a favorable choice for drug delivery due to its mucoadhesive properties and its ability to enhance permeation with sustained drug release [79]. This may explain the comparable improvement of productive performance in RNP-co-treated birds than birds supplemented with rutin alone following Cd exposure which coincided with [80] who confirmed that rutin nanoparticles displayed a notable enhancement in the antioxidant properties than rutin alone.

Regarding the economic evaluation, our findings indicated that exposure to Cd in broilers triggers significant economic losses. Our data is consistent with [81] who demonstrated that Cd-contaminated feed could accumulate in tissues and disrupt the metabolic and physiological functions which evoked higher mortality rates and ultimately influence the economic efficiency. Furthermore [82], revealed that dietary Cd supplementation at a dose level of 120 ppm had a critical impact on broiler performance, health parameters, and economic efficiency.

Our findings exhibited a relative increase in feed cost and TC in rutin and RNP-co-treated groups which may be attributed to the additional cost of rutin in diets. This finding aligns with [83] who observed that incorporating herbal additives into broiler diets raised the production costs of broilers. However, from an economic point of view, optimizing feed efficiency, enhancing FCR, and managing the stress risk effectively are essential for minimizing the production expenses and providing a financial benefit through enhancing the live BWG [84]. This has elevated the quest for cost-effective feed additive that could enhance the productivity via preserving gut health [85].

Improvement in return economic parameters, such as TR and NP, after co-treatment with rutin and RNPs was attributed to enhanced feed utilization, increased BWG, and improved FCR since rutin is regarded as a natural feed additive that could enhance feed efficiency via promoting digestive secretions and nutrient absorption, maintaining gut health, exhibiting antioxidant properties, and reducing the microbial load on the animal immune system [86–88]. Moreover, the additional costs of incorporating rutin or RNPs into the broilers’ diet were found to be negligible. Interestingly, broilers co-treated with RNPs showed a greater improvement in economic performance compared to the rutin co-treated group which may be attributed to the favorable effects of chitosan NP encapsulation on feed utilization, nutrient digestibility, growth performance, productivity, and immune responses [89, 90].

Overproduction of reactive oxygen species (ROS) resulted in compromising the antioxidant defense system including enzymatic biomarkers such as SOD and GPx which leads to oxidative stress [91]. The current study showed that the Cd-intoxicated group significantly raised MDA level with a marked reduction in antioxidant biomarkers such as SOD, GSH, CAT, and GPx in the BF tissue. These findings coincided with [92] who reported that dietary intake of Cd at a dose level of 100 mg/kg diet resulted in a significant elevation of MDA in the BF tissue, and also [93], displayed that a broiler diet containing Cd at a dose of 140 mg/kg provoked marked oxidative damage in the BF tissue with a notable decline in SOD and GPx. The ability of Cd to trigger oxidative damage may be due to its high affinity to thiol groups in the antioxidant enzymes with subsequent malfunctioning of their activity [94], and also, inhibition of NADPH oxidase is described as a possible mechanism of Cd-induced oxidative damage [95]. Furthermore, Cd could induce substantial alterations in the mitochondrial structure and mitochondrial permeability via blockage of the respiratory chain complexes [96].

Alternatively, rutin and RNP supplementation significantly raised the antioxidant enzyme activity with a marked reduction in MDA level in the BF tissue. These results come in agreement with [97] who reported that rutin at a dose level of 500 mg/kg diet could counteract oxidative damage via suppressing the MAPK pathway in the liver tissue of broilers. Rutin, also a well-known antioxidant, could boost the intestinal antioxidant status of laying hens exposed to oxidized protein of soybean meal [98]. Return function provides important insights into the impact of oxidative stress on the overall outcome of the production process since oxidative damage could provoke various pathophysiological changes in birds’ immune organs at the cellular level which is further associated with increased susceptibility to pathogens, compromised gut health, and reduced feed intake, weight gain, and economic efficiency [99]. Thus, our findings revealed that Cd-induced oxidative stress has a detrimental effect on broiler farm productivity and TR. In contrast, rutin and RNPs mitigated Cd-induced oxidative stress through enhancing the antioxidant enzyme activity that acts as ROS scavengers resulting in improved performance and return rates [100]. These findings are aligned with [37], who concluded that rutin supplementation could alleviate oxidative damage and improve productivity and TR in broilers.

Oxidative damage triggers excessive production of pro-inflammatory cytokines and CORT in broilers which is considered an important biomarker of stress and tissue damage in immune organs, particularly BF. The current study displayed that Cd-intoxicated birds displayed marked elevation of serum IL-6, TNF-α, and CORT hormone. Indeed, Cd could substantially elevate the expression levels of cytokines including IL-6 and TNF-α in central immune organs of poisoned chickens via the Toll-like receptor 4 (TLR4) pathway [7]. Moreover [93], revealed that Cd induced a complex inflammatory response in chicken splenic lymphocytes with significant elevation of various cytokines through upregulation of NF-κB expression level which appeared as a detrimental factor prompting the cytokine expression [101].

CORT or stress hormone is the main moderator of allostasis in broilers which is released following exposure to stressful conditions through stimulation of hypothalamic–pituitary–adrenal (HPA) axis resulting in numerous physiological changes to restore homeostasis [102]. Previous reports suggested that Cd triggers corticosteroid-induced immuno-regulatory circuit via activation of the HPA axis besides upregulation of glucocorticoid receptor (GR) expression, leading to a substantial rise in the glucocorticoid levels [103, 104]. Elevation of CORT hormone in broilers has a negative influence upon the hematopoietic system, particularly the TLC and differential leukocytic count, driving the dissolution of lymphocytes and inhibiting neutrophil apoptosis [105, 106]. The current study revealed that the Cd-intoxicated group showed a notable increase in the heterophil percentage and H/L ratio with a reduction of the lymphocyte percentage which may be due to the glucocorticoid influence. Similar findings were reported by [107] who concluded that Cd has marked immuno-toxic effects on the hematopoietic stem cells via increasing the myeloid progenitors and decreasing the lymphoid progenitors in exposed quails. Furthermore [108], documented that broilers experienced thermal stress-released excessive glucocorticoids resulting in severe leukopenia and lymphopenia with marked shift in the H/L ratio which regarded as an index of stress in broilers.

On contrary, rutin-co-treated birds showed a marked reduction of serum IL-6, TNF-α, and CORT hormone while RNP-co-treated broilers could efficiently restore the normal levels of such biomarkers. Similar findings from previous studies revealed that rutin had potent anti-inflammatory properties because of its inhibitory role in cytokine production, including TNF-α and IL6 [109, 110]. Furthermore, rutin effectively improved immunity and intestinal function via reducing pro-inflammatory cytokine content through inhibiting the Nrf2/HO-1 pathway in broilers [66, 111]. Additionally, our data revealed an increase in the lymphocytic population with a relative reduction of H/L ratio and heterophil percentage into normal levels. These findings coincided with [112, 113] who confirmed the immunomodulatory effect of rutin on hematopoietic tissue which may attributed to potent anti-oxidative potential of rutin. Furthermore, they hypothesized the capability of rutin to normalize the CORT homeostasis and HPA axis function following exposure to psychosocial stressful conditions which subsequently improve immunity and hematopoietic system [114].

Heat shock proteins (Hsp) are a part of the protein folding system in cells that are expressed intracellularly in all living organisms as a response to physiological stress, so they are regarded as stress-inducible and immunomodulants as they prompt the immune system response to adverse cellular conditions [115]. Notably, HSP70 plays an important role in proteostasis but, during stress, may be released to the extracellular matrix and triggers the pro-inflammatory response of immune cells which served as a hazard signal to the immune system [116, 117]. Extracellular Hsp70 initiates signal transduction facilitating the entry of NF-κB into the nucleus where it enhances the transcription of various pro-inflammatory cytokines as TNF-α and IL-6 [118]. Moreover, HSP70 can also trigger inflammatory responses via binding to TLRs, particularly TLR4, which play a vital role in the host innate immunity as a pattern recognition receptor superfamily [119, 120]. TLR4 can further activate various downstream inflammatory cascades such as the NF-κB pathway which results in the further production of various pro-inflammatory cytokines and chemokines [121, 122]. In the present study, our molecular and immunohistochemical findings revealed that the Cd-intoxicated group markedly upregulates the expression level of HSP70, TLR4, and NF-κB. These findings come in agreement with [123] who reported that Cd significantly promoted the expression level of the TLR4/NF-κB pathway in duck embryo hepatocytes, and also [124], exhibited that Cd enhanced the expression of HMGB1 downstream mediators, including TLR-4 and NF-κB which exacerbated the damaging effects of Cd in exposed tissues via stimulating the release of several inflammatory cytokines. Interestingly [125], revealed that Cd could induce adrenal damage through activation of TLR4/NF-κB-mediated inflammatory responses which play an essential role in Cd-induced toxic lesions. Moreover [126], elucidated that Cd triggered oxidative damage in the hepatic tissue through a substantial elevation of NF-κB and HSP70 expression levels.

On the other hand, rutin and RNP-co-treated birds displayed a prominent decline in HSP70, TLR4, and NF-κB expression levels which suggested that the HSP70/TLR4/NF-κB pathway may be responsible for improving the anti-inflammatory properties of RNPs. Our data matched with previous studies which illustrated that rutin downregulated the MAPK/HSPs/NF-κB pathway as a protective mechanism against induced necroptosis in liver [36, 127]. Furthermore, rutin played an immuno-regulatory role and protected the immune organs through TLR4/MyD88/NF-κB signaling pathway against cyclophosphamide-induced immunological stress [128]. Therefore, this pathway was suggested to play a vital role in not only inflammation but also the immune regulation, tissue repair, and survival of cells.

Overproduction of ROS and pro-inflammatory mediators considered potent signal molecules plays a pivotal role in mitochondrial-dependent apoptosis through enhancing the release of pro-apoptotic molecules causing caspase cascade activation and induction of apoptosis [129]. The Bcl2 family, of which Bcl-2 and Bax are the most important members, plays an essential role in the regulation of apoptosis via controlling the outer mitochondrial membrane permeability [130]. The current study exhibited a marked elevation of the mRNA and protein expression levels of pro-apoptotic caspase 3 and Bax in Cd-intoxicated broilers besides a significant decline of the anti-apoptotic Bcl-2 expression levels. These findings confirmed that Cd could exacerbate apoptosis and tissue damage in immune organs and subsequently deteriorate organ function which coincided with previous studies [131, 132] which illustrated that Cd could induce apoptosis and tissue damage at a dose level of 150 mg/kg diet in chicken BF tissue. Furthermore, increased mRNA level of Bax, caspase-3, and cytochrome c and decreased Bcl-2 and CaM was observed following Cd exposure in chicken splenic lymphocytes [133]. In opposition, rutin and particularly RNP-co-treated birds showed a marked downregulation of the mRNA and protein expression levels of caspase 3 and Bax with a significant rise of the anti-apoptotic Bcl-2 level. Our data come in agreement with [134] who reported that rutin acts as a scavenger of ROS with a potent anti-apoptotic effect at a dose level of 50 mg/kg BW in liver and kidney of rats exposed to deltamethrin. Moreover, [111] reported that rutin boosted cell proliferation and suppressed apoptosis in laying hens through Nrf2/HO-1 pathway.

Morphological examination revealed that Cd-intoxicated birds showed excessive damage of the BF tissue with marked lymphopenia and follicular atrophy which agreed with [16] who showed that Cd exposure at a dose level of 100 ppm could provoke severe atrophic changes in the lymphoid follicles with cellular edema since excessive ROS production in the BF tissue promotes a cascade of tissue damage, apoptosis, and impaired lymphocytic proliferation with a marked reduction of the B-lymphocytes [135]. On the other hand, RNP-co-treated groups retained the normal medullar and cortical morphology of the BF tissue which confirmed the immunoprotective effect of RNPs based on its antioxidant and anti-inflammatory properties which matched with previous studies [112, 136]. However, further investigation of Cd immunotoxicity and RNP immunomodulatory mechanisms through Western blotting will be required in the follow-up studies and regarded as one of the limitations in this study. Additionally, our study design could not give information about the relationship between Cd bioaccumulation in the bursal tissue and RNPs, and if RNPs could reduce Cd bioaccumulation in the bursal tissue, so further investigations are needed to clarify this since we thought that RNPs may be a promising candidate in adsorption of Cd.

## Conclusion

Cd treatment led to a severe decline in the productive performance of intoxicated broilers with marked economic losses. These findings were potentiated by marked oxidative damage in the BF tissue with a significant decline in the enzymatic and non-enzymatic antioxidant biomarkers such as CAT, SOD, GPx, and GSH with a substantial rise of MDA level which trigger excessive production of pro-inflammatory cytokines beside elevation of CORT level which in turn could rise the H/L ratio. Furthermore, the expression level of TLR4, HSP70, caspase3, NF-κB, and the pro-apoptotic Bax was significantly upregulated while the anti-apoptotic Bcl2 was downregulated in Cd-treated birds which suggested that HSP70/TLR4/NF-κB signaling pathway may be involved in the molecular mechanism of Cd immunotoxicity which is confirmed by the excessive damage of the BF tissue with marked lymphopenia, vacuolation, and follicular atrophy. Meanwhile, rutin and RNPs alleviated Cd-induced oxidative damage, suppressed release of pro-inflammatory cytokines, and modulated HSP70/TLR4/NF-κB molecular pathway which improved the morphological structure of the BF tissue. Our results also emphasized that RNPs were more effective against Cd-induced immunotoxicity than rutin alone since our nanoformulation could enhance solubility, bioavailability, targetability, and efficacy of rutin. Thus, further investigations are still needed to demonstrate other potential action mechanisms and safety of RNPs in broiler diets to both broiler and human health.

## Supplementary Information

Below is the link to the electronic supplementary material.

Supplementary file1 (TIF 1961 KB)

## Data Availability

No datasets were generated or analysed during the current study.

## References

[1] de Oliveira TF, Rossi EM, da Costa CS, Graceli JB, Krause M, Carneiro MTWD, Almenara CCP, Padilha AS (2023) Sex-dependent vascular effects of cadmium sub-chronic exposure on rats. Biometals 36:189–199 36418808 10.1007/s10534-022-00470-w

[2] Saedi S, Watson SE, Young JL, Tan Y, Wintergerst KA, Cai L (2023) Does maternal low-dose cadmium exposure increase the risk of offspring to develop metabolic syndrome and/or type 2 diabetes? Life Sci 315:121385 10.1016/j.lfs.2023.121385 PMC9912173 36634865

[3] Ali S, Bashir S, Mumtaz S, Shakir HA, Ara C, Ahmad F, Tahir HM, Faheem M, Irfan M, Masih A (2021) Evaluation of cadmium chloride-induced toxicity in chicks via hematological, biochemical parameters, and cadmium level in tissues. Biol Trace Elem Res 199:3457–3469 33125667 10.1007/s12011-020-02453-9

[4] Zhu Y, Guan H, Zhu X, Cai J, Jiao X, Shan J, Li Y, Wu Q, Zhang Z (2024) Astilbin antagonizes developmental cardiotoxicity after cadmium exposure in chicken embryos by inhibiting endoplasmic reticulum stress and maintaining calcium homeostasis. Ecotoxicol Environ Saf 270:115847 38118333 10.1016/j.ecoenv.2023.115847

[5] Zhang R, Yi R, Bi Y, Xing L, Bao J, Li J (2017) The effect of selenium on the Cd-induced apoptosis via NO-mediated mitochondrial apoptosis pathway in chicken liver. Biol Trace Elem Res 178:310–319 28062951 10.1007/s12011-016-0925-7

[6] Zhang R, Wang Y, Wang C, Zhao P, Liu H, Li J, Bao J (2017) Ameliorative effects of dietary selenium against cadmium toxicity is related to changes in trace elements in chicken kidneys. Biol Trace Elem Res 176:391–400 27561294 10.1007/s12011-016-0825-x

[7] Liu L-l, Zhang J-l, Zhang Z-w, Yao H-d, Sun G, Xu S-w (2014) Protective roles of selenium on nitric oxide-mediated apoptosis of immune organs induced by cadmium in chickens. Biol Trace Elem Res 159:199–209 24839000 10.1007/s12011-014-0007-7

[8] Tao C, Zhang B, Wei X, Zhao M, Sun Z, Wang S, Bi J, Qi D, Sun L, Zhang N (2020) Effects of dietary cadmium supplementation on production performance, cadmium residue in eggs, and hepatic damage in laying hens. Environ Sci Pollut Res 27:33103–33111 10.1007/s11356-020-09496-4 32529616

[9] Li J-L, Jiang C-Y, Li S, Xu S-W (2013) Cadmium induced hepatotoxicity in chickens (Gallus domesticus) and ameliorative effect by selenium. Ecotoxicol Environ Saf 96:103–109 23906702 10.1016/j.ecoenv.2013.07.007

[10] Aleksandrov AP, Mirkov I, Tucovic D, Kulas J, Zeljkovic M, Popovic D, Ninkov M, Jankovic S, Kataranovski M (2021) Immunomodulation by heavy metals as a contributing factor to inflammatory diseases and autoimmune reactions: cadmium as an example. Immunol Lett 240:106–122 34688722 10.1016/j.imlet.2021.10.003

[11] Höckner M, Piechnik CA, Fiechtner B, Weinberger B, Tomanek L (2020) Cadmium-related effects on cellular immunity comprises altered metabolism in earthworm coelomocytes. Int J Mol Sci 21:599 31963425 10.3390/ijms21020599 PMC7013597

[12] Wang Z, Sun Y, Yao W, Ba Q, Wang H (2021) Effects of cadmium exposure on the immune system and immunoregulation. Front Immunol 12:695484 34354707 10.3389/fimmu.2021.695484 PMC8330548

[13] Mirkov I, Aleksandrov AP, Ninkov M, Tucovic D, Kulas J, Zeljkovic M, Popovic D, Kataranovski M (2021) Immunotoxicology of cadmium: cells of the immune system as targets and effectors of cadmium toxicity. Food Chem Toxicol 149:112026 33508420 10.1016/j.fct.2021.112026

[14] Li N, Yi B-J, Saleem MAU, Li X-N, Li J-L (2023) Autophagy protects against Cd-induced cell damage in primary chicken hepatocytes via mitigation of oxidative stress and endoplasmic reticulum stress. Ecotoxicol Environ Saf 259:115056 37229871 10.1016/j.ecoenv.2023.115056

[15] Zhu M, Li H, Bai L, Wang L, Zou X (2020) Histological changes, lipid metabolism, and oxidative and endoplasmic reticulum stress in the liver of laying hens exposed to cadmium concentrations. Poult Sci 99:3215–3228 32475458 10.1016/j.psj.2019.12.073 PMC7597684

[16] Vayeghan A, Gharagozlou M, Broujeni G, Amoli J, Bokaee S, Hesaraki S (2011) The effect of different levels of cadmium on the histopathological changes and the rate of lymphoid cells apoptosis of bursa of Fabricius in broiler chickens. J Vet Res 66:193–277

[17] Vasiljeva S, Basova N, Smirnova G (2018) Disturbance of the functionality in immunocompetent organs of chickens due to accumulation of cadmium. Res Rural Dev 1:222–226

[18] Wang N, Jiang M, Zhang P, Shu H, Li Y, Guo Z, Li Y (2020) Amelioration of Cd-induced bioaccumulation, oxidative stress and intestinal microbiota by Bacillus cereus in Carassius auratus gibelio. Chemosphere 245:125613 31864061 10.1016/j.chemosphere.2019.125613

[19] Negahdari R, Bohlouli S, Sharifi S, Maleki Dizaj S, Rahbar Saadat Y, Khezri K, Jafari S, Ahmadian E, Gorbani Jahandizi N, Raeesi S (2021) Therapeutic benefits of rutin and its nanoformulations. Phytother Res 35:1719–1738 33058407 10.1002/ptr.6904

[20] Caglayan C, Kandemir FM, Yildirim S, Kucukler S, Eser G (2019) Rutin protects mercuric chloride-induced nephrotoxicity via targeting of aquaporin 1 level, oxidative stress, apoptosis and inflammation in rats. J Trace Elem Med Biol 54:69–78 31109623 10.1016/j.jtemb.2019.04.007

[21] Oboh G, Adebayo AA, Ademosun AO, Olowokere OG (2020) Rutin restores neurobehavioral deficits via alterations in cadmium bioavailability in the brain of rats exposed to cadmium. Neurotoxicology 77:12–19 31836556 10.1016/j.neuro.2019.12.008

[22] Imani A, Maleki N, Bohlouli S, Kouhsoltani M, Sharifi S, Maleki Dizaj S (2021) Molecular mechanisms of anticancer effect of rutin. Phytother Res 35:2500–2513 33295678 10.1002/ptr.6977

[23] Liu Y, Zhao X, Zhang Q, Wang L, Li Y, Li Y (2020) Characterization and evaluation of the solubility and oral bioavailability of rutin–ethanolate solvate. AAPS PharmSciTech 21:1–12 10.1208/s12249-020-01779-w 32839899

[24] Deepika MS, Thangam R, Sheena TS, Sasirekha R, Sivasubramanian S, Babu MD, Jeganathan K, Thirumurugan R (2019) A novel rutin-fucoidan complex based phytotherapy for cervical cancer through achieving enhanced bioavailability and cancer cell apoptosis. Biomed Pharmacother 109:1181–1195 30551368 10.1016/j.biopha.2018.10.178

[25] Gera S, Pooladanda V, Godugu C, Swamy Challa V, Wankar J, Dodoala S, Sampathi S (2020) Rutin nanosuspension for potential management of osteoporosis: effect of particle size reduction on oral bioavailability, in vitro and in vivo activity. Pharm Dev Technol 25:971–988 32403972 10.1080/10837450.2020.1765378

[26] Ramaswamy S, Dwarampudi LP, Kadiyala M, Kuppuswamy G, Reddy KVVS, Kumar CKA, Paranjothy M (2017) Formulation and characterization of chitosan encapsulated phytoconstituents of curcumin and rutin nanoparticles. Int J Biol Macromol 104:1807–1812 28668610 10.1016/j.ijbiomac.2017.06.112

[27] Aluani D, Tzankova V, Kondeva-Burdina M, Yordanov Y, Nikolova E, Odzhakov F, Apostolov A, Markova T, Yoncheva K (2017) Evaluation of biocompatibility and antioxidant efficiency of chitosan-alginate nanoparticles loaded with quercetin. Int J Biol Macromol 103:771–782 28536020 10.1016/j.ijbiomac.2017.05.062

[28] Palei NN, Surendran V, Ramaswamy S, Ravula P (2023) Estimation of rutin-loaded chitosan sodium alginate nanoparticles in rat plasma using a chemometrics-assisted bioanalytical high-performance liquid chromatography method. Separation Sci Plus 6:2200138

[29] Mujtaba MA, Hassan KA, Imran M (2018) Chitosan-alginate nanoparticles as a novel drug delivery system for rutin. Int J Adv Biotechnol Res 9:1895–1903

[30] Patil AG, Jobanputra AH, Engineering (2015) Rutin-chitosan nanoparticles: fabrication, characterization and application in dental disorders. Polymer-Plastics Technol 54:202–208

[31] Chang C, Zhang L, Miao Y, Fang B, Yang Z (2021) Anticancer and apoptotic-inducing effects of rutin-chitosan nanoconjugates in triple negative breast cancer cells. J Cluster Sci 32:331–340

[32] Sorasitthiyanukarn FN, Muangnoi C, Bhuket PRN, Rojsitthisak P, Rojsitthisak P (2018) Chitosan/alginate nanoparticles as a promising approach for oral delivery of curcumin diglutaric acid for cancer treatment. Mater Sci Eng C 93:178–190 10.1016/j.msec.2018.07.069 30274050

[33] José-Yacamán M, Marın-Almazo M, Ascencio J (2001) High resolution TEM studies on palladium nanoparticles. J Mol Catal A: Chem 173:61–74

[34] Dubey R, Bajpai J, Bajpai A (2016) Chitosan-alginate nanoparticles (CANPs) as potential nanosorbent for removal of Hg (II) ions. Environ Nanotechnol Monit Manag 6:32–44

[35] Dale N (1994) National Research Council nutrient requirements of poultry–ninth revised edition (1994). J Appl Poultry Res 3: 101

[36] Rahmani S, Naraki K, Roohbakhsh A, Hayes AW, Karimi G (2023) The protective effects of rutin on the liver, kidneys, and heart by counteracting organ toxicity caused by synthetic and natural compounds. Food Sci Nutr 11:39–56 36655104 10.1002/fsn3.3041 PMC9834893

[37] Wahed NM, Abomosallam M, Hendam BM, Shouman Z, Hashem N, Sakr SA (2023) Economic and productive comparison of rutin and rutin-loaded chitosan alginate nanoparticles against lead-induced oxidative stress in Cobb and arbor broiler breeds. Biol Trace Element Res: 1–20 10.1007/s12011-023-04019-x PMC11338976 38153670

[38] Xing L, Zhang R, Gong R, Liu X, Bao J, Li J (2022) Ameliorative effects of dietary selenium against cadmium toxicity on production performance and egg quality in laying hens. Ecotoxicol Environ Saf 248:114317 36435000 10.1016/j.ecoenv.2022.114317

[39] Zhang R, Liu Y, Xing L, Zhao N, Zheng Q, Li J, Bao J (2018) The protective role of selenium against cadmium-induced hepatotoxicity in laying hens: expression of Hsps and inflammation-related genes and modulation of elements homeostasis. Ecotoxicol Environ Saf 159:205–212 29753822 10.1016/j.ecoenv.2018.05.016

[40] Jaksch W (1981) Euthanasia of day-old male chicks in the poultry industry. Int J Study Anim Problems 2:8

[41] Alagawany M, Abd El-Hack M, Farag M, Shaheen H, Abdel-Latif M, Noreldin A, Patra A (2018) The usefulness of oregano and its derivatives in poultry nutrition. Worlds Poult Sci J 74:463–474

[42] Awobajo O, Akinrolabu R, Mako A, Igbosanu A, Olatokunbo O (2007) The mortality rate of two different breeds of broilers after brooding stage to maturity. Middle-East J Sci Res 2:37–42

[43] Tareen MH, Wagan R, Siyal FA, Babazadeh D, Bhutto ZA, Arain MA, Saeed M (2017) Effect of various levels of date palm kernel on growth performance of broilers. Veterinary world 10:227 28344407 10.14202/vetworld.2017.227-232 PMC5352849

[44] Waheed N, Attalah S, Darwish R, Fouda M (2019) The effects of dried guava waste and dried olive cake as substitutes for alfalfa on rabbit farm profit. Mansoura Vet Med J 20:15–20

[45] Aziza AE, Ibrahim SS, Mahmoud R (2017) Growth performance and economic efficiency of growing New Zealand white rabbits fed different levels of crud fiber by using corn cob with and without enzyme supplementation. Alexandria J Vet Sci 55:99–109

[46] Omar MA (2019) Economic evaluation of using dried brewer’s yeast as feed additives for two broiler breeds. Damanhour J Vet Sci 3:8–11

[47] Yang Y, Yang L, Liu S (2020) Analysis of agricultural economic growth factors based on Cobb Douglas production. E3S Web Conf 189: 01002

[48] Wonnacott P, Wonnacott R (1981) Is unilateral tariff reduction preferable to a customs union? The curious case of the missing foreign tariffs. Am Econ Rev 71:704–714

[49] Rawson T, Colles FM, Smith AL, Dawkins MS, Bonsall MB (2021) Can good broiler flock welfare prevent colonization by Campylobacter? Poult Sci 100:101420 34607156 10.1016/j.psj.2021.101420 PMC8493578

[50] Keustermans GC, Hoeks SB, Meerding JM, Prakken BJ, de Jager W (2013) Cytokine assays: an assessment of the preparation and treatment of blood and tissue samples. Methods 61:10–17 23603216 10.1016/j.ymeth.2013.04.005

[51] Soria MA, Bonnet MA, Bueno DJ (2015) Relationship of Salmonella infection and inflammatory intestinal response with hematological and serum biochemical values in laying hens. Vet Immunol Immunopathol 165:145–153 25912484 10.1016/j.vetimm.2015.03.008

[52] Campbell TW (1995) Avian hematology and cytology. Iowa State University Press

[53] Livak KJ, Schmittgen TD (2001) Analysis of relative gene expression data using real-time quantitative PCR and the 2− ΔΔCT method. Methods 25:402–408 11846609 10.1006/meth.2001.1262

[54] Anderson G, Bancroft J (2002) Tissue processing and microtomy. Theory Pract Histol Techniques 5:85–99

[55] Shalaby AM, Bahey NG (2018) Reversal of the hepatic damage induced by the supraphysiological dose of nandrolone decanoate after its withdrawal in the adult male rat. Tissue and Cell 53:44–52 30060826 10.1016/j.tice.2018.05.013

[56] Ibm C (2012) IBM SPSS statistics for Windows. Armonk : IBM Corp

[57] Genchi G, Sinicropi MS, Lauria G, Carocci A, Catalano A (2020) The effects of cadmium toxicity. Int J Environ Res Public Health 17:3782 32466586 10.3390/ijerph17113782 PMC7312803

[58] Hamid Y, Tang L, Sohail MI, Cao X, Hussain B, Aziz MZ, Usman M, He Z-l, Yang X (2019) An explanation of soil amendments to reduce cadmium phytoavailability and transfer to food chain. Sci Total Environ 660:80–96 30639721 10.1016/j.scitotenv.2018.12.419

[59] Rigby H, Smith SR (2020) The significance of cadmium entering the human food chain via livestock ingestion from the agricultural use of biosolids, with special reference to the UK. Environ Int 143:105844 32673905 10.1016/j.envint.2020.105844

[60] Khafaga AF, Abd El-Hack ME, Taha AE, Elnesr SS, Alagawany M (2019) The potential modulatory role of herbal additives against Cd toxicity in human, animal, and poultry: a review. Environ Sci Pollut Res 26:4588–4604 10.1007/s11356-018-4037-0 30612355

[61] Li L, Cao Y, Ippolito JA, Xing W, Qiu K, Li H, Zhao D, Wang Y, Wang Y (2023) Cadmium and lead bioavailability to poultry fed with contaminated soil-spiked feed. Sci Total Environ 879:163036 36972887 10.1016/j.scitotenv.2023.163036

[62] Ceramella J, De Maio AC, Basile G, Facente A, Scali E, Andreu I, Sinicropi MS, Iacopetta D, Catalano A (2024) Phytochemicals involved in mitigating silent toxicity induced by heavy metals. Foods 13:978 38611284 10.3390/foods13070978 PMC11012104

[63] Hassan FA, Roushdy EM, Kishawy AT, Zaglool AW, Tukur HA, Saadeldin IM (2018) Growth performance, antioxidant capacity, lipid-related transcript expression and the economics of broiler chickens fed different levels of rutin. Animals 9:7 30583506 10.3390/ani9010007 PMC6357029

[64] Truzzi F, Tibaldi C, Zhang Y, Dinelli G, D Amen E (2021) An overview on dietary polyphenols and their biopharmaceutical classification system (BCS). Int J Mol Sci 22:5514 34073709 10.3390/ijms22115514 PMC8197262

[65] Han X, Zhang M, Zhang R, Huang L, Jia X, Huang F, Liu L (2020) Physicochemical interactions between rice starch and different polyphenols and structural characterization of their complexes. Lwt 125:109227

[66] Chen S, Liu H, Zhang J, Zhou B, Zhuang S, He X, Wang T, Wang C (2022) Effects of different levels of rutin on growth performance, immunity, intestinal barrier and antioxidant capacity of broilers. Ital J Anim Sci 21:1390–1401

[67] Friedman AJ, Phan J, Schairer DO, Champer J, Qin M, Pirouz A, Blecher-Paz K, Oren A, Liu PT, Modlin RL (2013) Antimicrobial and anti-inflammatory activity of chitosan–alginate nanoparticles: a targeted therapy for cutaneous pathogens. J Investig Dermatol 133:1231–1239 23190896 10.1038/jid.2012.399 PMC3631294

[68] Ahmad N, Ahmad R, Naqvi AA, Alam MA, Ashafaq M, Samim M, Iqbal Z, Ahmad FJ (2016) Rutin-encapsulated chitosan nanoparticles targeted to the brain in the treatment of cerebral ischemia. Int J Biol Macromol 91:640–655 27264648 10.1016/j.ijbiomac.2016.06.001

[69] Surendran V, Palei NN (2022) Formulation and characterization of rutin loaded chitosan-alginate nanoparticles: antidiabetic and cytotoxicity studies. Curr Drug Deliv 19:379–394 34636298 10.2174/1567201818666211005090656

[70] Al-Waeli A, Zoidis E, Pappas A, Demiris N, Zervas G, Fegeros K (2013) The role of organic selenium in cadmium toxicity: effects on broiler performance and health status. Animal 7:386–393 23031417 10.1017/S1751731112001590

[71] Erdogan Z, Erdogan S, Celik S, Unlu A (2005) Effects of ascorbic acid on cadmium-induced oxidative stress and performance of broilers. Biol Trace Elem Res 104:19–31 15851829 10.1385/BTER:104:1:019

[72] Patel UD, Bhatt P, Pandya K, Patel H, Modi C (2021) Cadmium induced oxidative stress-mediated pathophysiological alterations in chickens and their amelioration by polyherbal mixture enriched feed. Indian J Traditional Knowledge (IJTK) 20:41–53

[73] Berbesh S, El-Shawarby R, El-Shewy E, El-Sheshtawy S, Elshafae S (2022) Ameliorative effect of spirulina platensis against cadmium toxicity in broiler chickens. Benha Vet Med J 42:51–55

[74] Goliomytis M, Orfanou H, Petrou E, Charismiadou M, Simitzis P, Deligeorgis S (2014) Effect of hesperidin dietary supplementation on hen performance, egg quality and yolk oxidative stability. Br Poult Sci 55:98–104 24397432 10.1080/00071668.2013.870328

[75] Daramola OT (2019) Medicinal plants leaf meal supplementation in broiler chicken diet: effects on performance characteristics, serum metabolite and antioxidant status. Anim Res Int 16:3334–3342

[76] Ahmadipour B, Hassanpour H, Khajali F (2018) Evaluation of hepatic lipogenesis and antioxidant status of broiler chickens fed mountain celery. BMC Vet Res 14:1–7 30103743 10.1186/s12917-018-1561-6 PMC6088407

[77] Prihambodo TR, Sholikin MM, Qomariyah N, Jayanegara A, Batubara I, Utomo DB, Nahrowi N (2021) Effects of dietary flavonoids on performance, blood constituents, carcass composition and small intestinal morphology of broilers: a meta-analysis. Anim Biosci 34:434 32898948 10.5713/ajas.20.0379 PMC7961189

[78] Dube A, Nicolazzo JA, Larson I (2010) Chitosan nanoparticles enhance the intestinal absorption of the green tea catechins (+)-catechin and (−)-epigallocatechin gallate. Eur J Pharm Sci 41:219–225 20600878 10.1016/j.ejps.2010.06.010

[79] George M, Abraham TE (2006) Polyionic hydrocolloids for the intestinal delivery of protein drugs: alginate and chitosan—a review. J Control Release 114:1–14 16828914 10.1016/j.jconrel.2006.04.017

[80] Remanan MK, Zhu F (2021) Encapsulation of rutin using quinoa and maize starch nanoparticles. Food Chem 353:128534 33189475 10.1016/j.foodchem.2020.128534

[81] Olgun O, Yildiz A, Şahin A (2020) Evaluation of dietary presence or use of cadmium in poultry. Worlds Poult Sci J 76:64–73

[82] El-Dein A, Galal A, Attia M, El-Motaal A (2000) Effect of dietary cadmium supplementation on broiler performance and economic efficiency. Egypt Poultry Sci J 20:295–310

[83] Karangiya V, Savsani H, Patil SS, Garg D, Murthy K, Ribadiya N, Vekariya S (2016) Effect of dietary supplementation of garlic, ginger and their combination on feed intake, growth performance and economics in commercial broilers. Vet World 9:245 27057106 10.14202/vetworld.2016.245-250 PMC4823283

[84] Fu C, Shah AA, Khan RU, Khan MS, Wanapat M (2023) Emerging trends and applications in health-boosting microorganisms-specific strains for enhancing animal health. Microb Pathog 183:106290 10.1016/j.micpath.2023.106290 37567325

[85] Upadhayay U, Vishwa PCV (2014) Growth promoters and novel feed additives improving poultry production and health, bioactive principles and beneficial applications: the trends and advances-a review. Int J Pharmacol 10:129–159

[86] Abdelli N, Solà-Oriol D, Pérez JF (2021) Phytogenic feed additives in poultry: achievements, prospective and challenges. Animals 11:3471 34944248 10.3390/ani11123471 PMC8698016

[87] Omar JA, Hejazi A, Badran R (2016) Performance of broilers supplemented with natural herb extract. Open J Anim Sci 6:68–74

[88] Singh V, Singh V, Dwivedi D, Tiwari D, Singh S, Singh V (2018) Effect of a phytogenic feed additive supplemented diet on growth performance, hemato-biochemical profile and carcass characteristics of broiler chickens. Anim Nutr Feed Technol 18:321–331

[89] Hua S, Marks E, Schneider JJ, Keely S (2015) Advances in oral nano-delivery systems for colon targeted drug delivery in inflammatory bowel disease: selective targeting to diseased versus healthy tissue. Nanomed: Nanotechnol Biol Med 11: 1117–1132 10.1016/j.nano.2015.02.018 25784453

[90] Huang R, Yin Y, Wu G, Zhang Y, Li T, Li L, Li M, Tang Z, Zhang J, Wang B (2005) Effect of dietary oligochitosan supplementation on ileal digestibility of nutrients and performance in broilers. Poult Sci 84:1383–1388 16206559 10.1093/ps/84.9.1383

[91] Jiao X, Yang K, An Y, Teng X, Teng X (2017) Alleviation of lead-induced oxidative stress and immune damage by selenium in chicken bursa of Fabricius. Environ Sci Pollut Res 24:7555–7564 10.1007/s11356-016-8329-y 28116627

[92] Bharavi K, Reddy AG, Rao G, Reddy AR, Rao SR (2010) Reversal of cadmium-induced oxidative stress in chicken by herbal adaptogens Withania somnifera and Ocimum sanctum. Toxicol Int 17:59 21170246 10.4103/0971-6580.72671 PMC2997456

[93] Li R, Zhang L, Tang Z, Li T, Li G, Zhang R, Ge M (2019) Effects of fungal polysaccharide on oxidative damage and TLR4 pathway to the central immune organs in cadmium intoxication in chickens. Biol Trace Elem Res 191:464–473 30632076 10.1007/s12011-018-1627-0

[94] Cuypers A, Plusquin M, Remans T, Jozefczak M, Keunen E, Gielen H, Opdenakker K, Nair AR, Munters E, Artois TJ (2010) Cadmium stress: an oxidative challenge. Biometals 23:927–940 20361350 10.1007/s10534-010-9329-x

[95] Kitamura M, Hiramatsu N (2010) The oxidative stress: endoplasmic reticulum stress axis in cadmium toxicity. Biometals 23:941–950 20130962 10.1007/s10534-010-9296-2

[96] Matović V, Buha A, Ðukić-Ćosić D, Bulat Z (2015) Insight into the oxidative stress induced by lead and/or cadmium in blood, liver and kidneys. Food Chem Toxicol 78:130–140 25681546 10.1016/j.fct.2015.02.011

[97] Liu L, Zhao L, Liu Y, Yu X, Qiao X (2022) Rutin ameliorates cadmium-induced necroptosis in the chicken liver via inhibiting oxidative stress and MAPK/NF-κB pathway. Biol Trace Elem Res 200:1799–1810 34091842 10.1007/s12011-021-02764-5

[98] Li H, Jin R, Gu Y, Zhou Y (2023) Effects of rutin supplementation on intestinal morphology, antioxidant capacity, immunity, and gut microbiota of laying hens fed a diet containing stored soybean meal. Ital J Anim Sci 22:1283–1293

[99] Ghanima MMA, Abd El-Hack ME, Othman SI, Taha AE, Allam AA, Abdel-Moneim A-ME (2020) Impact of different rearing systems on growth, carcass traits, oxidative stress biomarkers, and humoral immunity of broilers exposed to heat stress. Poult Sci 99:3070–3078 32475443 10.1016/j.psj.2020.03.011 PMC7597735

[100] Ponnampalam EN, Kiani A, Santhiravel S, Holman BW, Lauridsen C, Dunshea FR (2022) The importance of dietary antioxidants on oxidative stress, meat and milk production, and their preservative aspects in farm animals: antioxidant action, animal health, and product quality—Invited review. Animals 12:3279 36496798 10.3390/ani12233279 PMC9738477

[101] Chen Y, Yang B, Stanton C, Ross RP, Zhao J, Zhang H, Chen W (2021) Bifidobacterium pseudocatenulatum ameliorates DSS-induced colitis by maintaining intestinal mechanical barrier, blocking proinflammatory cytokines, inhibiting TLR4/NF-κB signaling, and altering gut microbiota. J Agric Food Chem 69:1496–1512 33512996 10.1021/acs.jafc.0c06329

[102] Zaytsoff SJ, Brown CL, Montina T, Metz GA, Abbott DW, Uwiera RR, Inglis GD (2019) Corticosterone-mediated physiological stress modulates hepatic lipid metabolism, metabolite profiles, and systemic responses in chickens. Sci Rep 9:19225 31848364 10.1038/s41598-019-52267-6 PMC6917734

[103] Zhang Q, Xu W, Kong Z, Wu Y, Liu Y (2023) Cadmium exposure-induced rat testicular dysfunction and its mechanism of chronic stress. Food Chem Toxicol 182:114181 10.1016/j.fct.2023.114181 37972751

[104] Chen N, Tong X, Wu S, Xu X, Chen Q, Wang F (2022) Cadmium induces placental glucocorticoid barrier damage by suppressing the cAMP/PKA/Sp1 pathway and the protective role of taurine. Toxicol Appl Pharmacol 440:115938 35219639 10.1016/j.taap.2022.115938

[105] Scanes CG (2016) Biology of stress in poultry with emphasis on glucocorticoids and the heterophil to lymphocyte ratio. Poult Sci 95:2208–2215 27252367 10.3382/ps/pew137

[106] Powolny T, Bassin N, Crini N, Fourel I, Morin C, Pottinger T, Massemin S, Zahn S, Coeurdassier M (2020) Corticosterone mediates telomere length in raptor chicks exposed to chemical mixture. Sci Total Environ 706:135083 31841853 10.1016/j.scitotenv.2019.135083

[107] Suljevic D, Corbic A, Islamagic E, Focak M, Filipic F, Alijagic A (2019) Impairments of bone marrow hematopoietic cells followed by the sever erythrocyte damage and necrotic liver as the outcome of chronic in vivo exposure to cadmium: novel insights from quails. Environ Toxicol Pharmacol Therapeutics 72:103250 10.1016/j.etap.2019.103250 31521044

[108] Roushdy EM, Zaglool AW, Hassan FA (2020) Thermal stress consequences on growth performance, immunological response, antioxidant status, and profitability of finishing broilers: transcriptomic profile change of stress-related genes. Trop Anim Health Prod 52:3685–3696 32978744 10.1007/s11250-020-02405-4

[109] Liu S, Adewole D, Yu L, Sid V, Wang B, Karmin O, Yang C (2019) Rutin attenuates inflammatory responses induced by lipopolysaccharide in an in vitro mouse muscle cell (C2C12) model. Poult Sci 98:2756–2764 30753670 10.3382/ps/pez037

[110] Awad A, Zaglool AW, Khalil SR (2018) Immunohaematological status and mRNA expression of the genes encoding interleukin-6, nuclear-factor kappa B, and tumor-necrosis factor-α in the spleen of broilers supplemented with dietary rutin. Anim Prod Sci 59:1454–1461

[111] Wu Y, Zhou S, Zhao A, Mi Y, Zhang C (2023) Protective effect of rutin on ferroptosis-induced oxidative stress in aging laying hens through Nrf2/HO-1 signaling. Cell Biol Int 47:598–611 36378583 10.1002/cbin.11960

[112] Ganeshpurkar A, Saluja AK (2017) Protective effect of rutin on humoral and cell mediated immunity in rat model. Chem Biol Interact 273:154–159 28606468 10.1016/j.cbi.2017.06.006

[113] Roy S, Majumdar S, Singh AK, Ghosh B, Ghosh N, Manna S, Chakraborty T, Mallick S (2015) Synthesis, characterization, antioxidant status, and toxicity study of vanadium–rutin complex in Balb/c mice. Biol Trace Elem Res 166:183–200 25697629 10.1007/s12011-015-0270-2

[114] Emudainohwo JO, Ben-Azu B, Adebayo OG, Aduema W, Uruaka C, Ajayi AM, Okpakpor EE, Ozolua RI (2023) Normalization of HPA axis, cholinergic neurotransmission, and inhibiting brain oxidative and inflammatory dynamics are associated with the adaptogenic-like effect of rutin against psychosocial defeat stress. J Mol Neurosci 73:60–75 36580190 10.1007/s12031-022-02084-w

[115] Zininga T, Ramatsui L, Shonhai A (2018) Heat shock proteins as immunomodulants. Molecules 23:2846 30388847 10.3390/molecules23112846 PMC6278532

[116] He S, Yu Q, He Y, Hu R, Xia S, He J (2019) Dietary resveratrol supplementation inhibits heat stress-induced high-activated innate immunity and inflammatory response in spleen of yellow-feather broilers. Poult Sci 98:6378–6387 31406997 10.3382/ps/pez471 PMC8913767

[117] Jiang S, Mohammed A, Jacobs J, Cramer T, Cheng H (2020) Effect of synbiotics on thyroid hormones, intestinal histomorphology, and heat shock protein 70 expression in broiler chickens reared under cyclic heat stress. Poult Sci 99:142–150 32416795 10.3382/ps/pez571 PMC7587863

[118] Feng Y, Yang S, Ma Y, Bai X-Y, Chen X (2015) Role of Toll-like receptors in diabetic renal lesions in a miniature pig model. Sci Adv 1:e1400183 26601192 10.1126/sciadv.1400183 PMC4640603

[119] Salama A, Asaad GF, Shaheen A (2022) Chrysin ameliorates STZ-induced diabetes in rats: possible impact of modulation of TLR4/NF-κβ pathway. Res Pharm Sci 17:1 34909039 10.4103/1735-5362.329921 PMC8621845

[120] Qu B, Jia Y, Liu Y, Wang H, Ren G, Wang H (2015) The detection and role of heat shock protein 70 in various nondisease conditions and disease conditions: a literature review. Cell Stress Chaperones 20:885–892 26139132 10.1007/s12192-015-0618-8 PMC4595429

[121] Zhao Z, Qu F, Liu R, Xia Y (2020) Differential expression of miR-142-3p protects cardiomyocytes from myocardial ischemia-reperfusion via TLR4/NFkB axis. J Cell Biochem 121:3679–3690 31746021 10.1002/jcb.29506

[122] Rashidian A, Muhammadnejad A, Dehpour A-R, Mehr SE, Akhavan MM, Shirkoohi R, Chamanara M, Mousavi S-E, Rezayat S-M (2016) Atorvastatin attenuates TNBS-induced rat colitis: the involvement of the TLR4/NF-kB signaling pathway. Inflammopharmacology 24:109–118 27038922 10.1007/s10787-016-0263-6

[123] Cao Z, Yang F, Lin Y, Shan J, Cao H, Zhang C, Zhuang Y, Xing C, Hu G (2022) Selenium antagonizes cadmium-induced inflammation and oxidative stress via suppressing the interplay between NLRP3 inflammasome and HMGB1/NF-κB pathway in duck hepatocytes. Int J Mol Sci 23:6252 35682929 10.3390/ijms23116252 PMC9181349

[124] Hafez HM, Waz S, El-Tahawy NFG, Mohamed MZ (2022) Agomelatine ameliorates cadmium-induced toxicity through the modification of HMGB-1/TLR-4/NFκB pathway. Toxicol Appl Pharmacol 457:116313 36356678 10.1016/j.taap.2022.116313

[125] Yao Y, Zhao X, Zheng S, Wang S, Liu H, Xu S (2021) Subacute cadmium exposure promotes M1 macrophage polarization through oxidative stress-evoked inflammatory response and induces porcine adrenal fibrosis. Toxicology 461:152899 34416349 10.1016/j.tox.2021.152899

[126] Farag MR, Alagawany M, Mahdy EA, El-Hady E, Abou-Zeid SM, Mawed SA, Azzam MM, Crescenzo G, Abo-Elmaaty AM (2023) Benefits of Chlorella vulgaris against cadmium chloride-induced hepatic and renal toxicities via restoring the cellular redox homeostasis and modulating Nrf2 and NF-KB pathways in male rats. Biomedicines 11:2414 37760855 10.3390/biomedicines11092414 PMC10525457

[127] Huang FM, Chang YC, Lee MW, Su NY, Yang LC, Kuan YH (2023) Rutin alleviates bisphenol A-glycidyl methacrylate-induced generation of proinflammatory mediators through the MAPK and NF-κB pathways in macrophages. Environ Toxicol 38:628–634 36413001 10.1002/tox.23711

[128] Lan Z, Wang H, Wang S, Zhu T, Ma S, Song Y, Cui C, Liu M, Tian C (2022) Rutin protects against cyclophosphamide induced immunological stress by inhibiting TLR4-NF-κB-mediated inflammation and activating the Nrf2-mediated antioxidant responses. Pharmacol Res-Modern Chinese Med 4:100135

[129] Fu S-C, Liu J-M, Lee K-I, Tang F-C, Fang K-M, Yang C-Y, Su C-C, Chen H-H, Hsu R-J, Chen Y-W (2020) Cr (VI) induces ROS-mediated mitochondrial-dependent apoptosis in neuronal cells via the activation of Akt/ERK/AMPK signaling pathway. Toxicol In Vitro 65:104795 32061800 10.1016/j.tiv.2020.104795

[130] Gao J, Tian X, Yan X, Wang Y, Wei J, Wang X, Yan X, Song G (2021) Selenium exerts protective effects against fluoride-induced apoptosis and oxidative stress and altered the expression of Bcl-2/caspase family. Biol Trace Elem Res 199:682–692 32613488 10.1007/s12011-020-02185-w

[131] Chen H, Li P, Shen Z, Wang J, Diao L (2021) Protective effects of selenium yeast against cadmium-induced necroptosis through mir-26a-5p/PTEN/PI3K/AKT signaling pathway in chicken kidney. Ecotoxicol Environ Saf 220:112387 34111659 10.1016/j.ecoenv.2021.112387

[132] Zhang D, Yang X-y, Qin Y-z, Wu G-d, Ning G-b, Huo N-r, Tian W-x (2020) Antagonistic effect of N-acetyl-L-cysteine against cadmium-induced cytotoxicity and abnormal immune response on chicken peritoneal macrophages. Ecotoxicol Environ Saf 206:111185 32890923 10.1016/j.ecoenv.2020.111185

[133] Guan T-Q, Qiu B-H, Nurmamedov H, Talukder M, Lv M-W, Li J-L (2022) Cadmium-induced splenic lymphocytes anoikis is not mitigated by activating Nrf2-mediated antioxidative defense response. J Inorg Biochem 234:111882 35752064 10.1016/j.jinorgbio.2022.111882

[134] Küçükler S, Kandemir FM, Özdemir S, Çomaklı S, Caglayan C (2021) Protective effects of rutin against deltamethrin-induced hepatotoxicity and nephrotoxicity in rats via regulation of oxidative stress, inflammation, and apoptosis. Environ Sci Pollut Res 28:62975–62990 10.1007/s11356-021-15190-w 34218375

[135] Chen K, Fang J, Peng X, Cui H, Chen J, Wang F, Chen Z, Zuo Z, Deng J, Lai W (2014) Effect of selenium supplementation on aflatoxin B1-induced histopathological lesions and apoptosis in bursa of Fabricius in broilers. Food Chem Toxicol 74:91–97 25261862 10.1016/j.fct.2014.09.003

[136] Hamad RS (2023) Rutin, a flavonoid compound derived from garlic, as a potential immunomodulatory and anti-inflammatory agent against murine Schistosomiasis mansoni. Nutrients 15:1206 36904204 10.3390/nu15051206 PMC10005531

