# Supplementary figures and images for: Rutin Nanoparticles Alleviate Cadmium-Induced Oxidative and Immune Damage in Broilers’ Bursa of Fabricius via Modulating Hsp70/TLR4/NF-κB Signaling Pathway
Source: Biol Trace Elem Res. 2024 May 4;203(2):1016–34. doi: 10.1007/s12011-024-04199-0 (PMC11750906; doi:10.1007/s12011-024-04199-0)

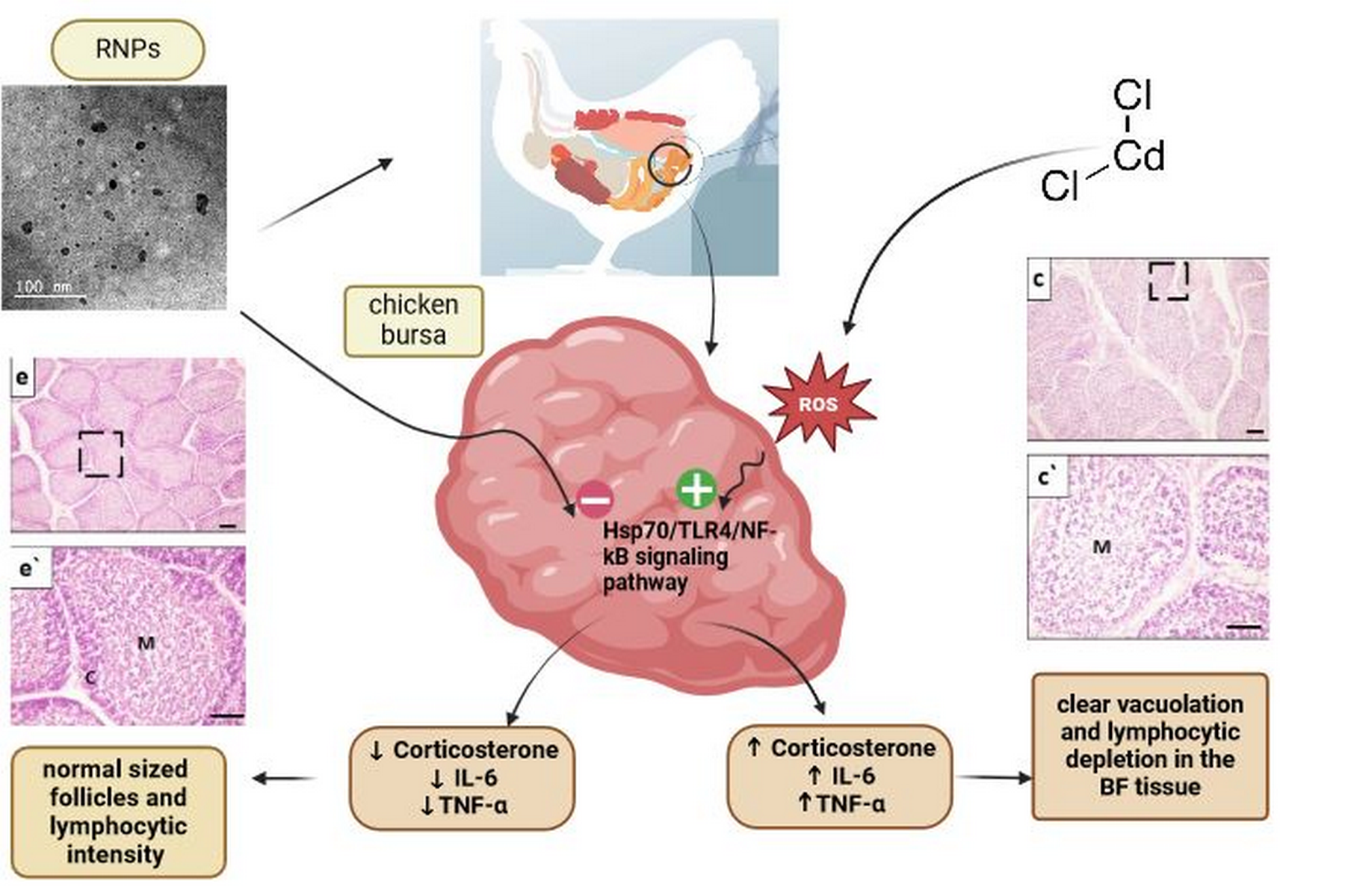

Supplement: Supplementary file 1 — Supplementary file1 (TIF 1961 KB) [file 12011_2024_4199_MOESM1_ESM.tif]
